# Combined multi-omics and multi-spectral profiling of plasma extracellular vesicles reveals liquid biopsy biomarkers for glioma diagnosis

**DOI:** 10.1016/j.xcrm.2026.102744

**Published:** 2026-04-17

**Authors:** Stephen David Robinson, Biniam Tsegay Haile, Matthew Reily-Bell, Olivia Iwanowytsch, Siobhan Palmer, Dorte Schou Nørøxe, Panagiota S. Filippou, Joanna Renaut, Alan Lazarus, Georgios Antoniou, Mark Samuels, Viviana Vella, Chrysa Filippopoulou, William Jones, Josephine Jung, Xiaoou Li, Nan Ji, Yang Zhang, Aleena Azam, Jane Skjoeth-Rasmussen, Ulrik Lassen, Adriana Saraiva, Ahmad Taha, Tania Slatter, Greg Jones, Rajesh Katare, Holly J. Butler, Matthew J. Baker, Marilena Hadjidemetriou, Duncan Gilbert, Benjamin Towler, Keyoumars Ashkan, Giles Critchley, Frances M.G. Pearl, Georgios Giamas

**Affiliations:** 1 International Oncology Institute, The First Affiliated Hospital of Zhejiang Chinese Medical University, Oncology Department of the First Affiliated Hospital of Zhejiang Chinese Medical University, Hangzhou 310053, China; 2 Department of Biochemistry and Biomedicine, School of Life Sciences, University of Sussex, Brighton BN1 9QG, UK; 3 Sussex Cancer Centre, University Hospitals Sussex NHS Foundation Trust, Brighton BN2 5BE, UK; 4 Bioinformatics Laboratory, School of Life Sciences, University of Sussex, Brighton BN1 9QG, UK; 5 Dunedin School of Medicine, University of Otago, Dunedin 9016, New Zealand; 6 NanoOmics Lab, Centre for Nanotechnology in Medicine, Faculty of Biology, Medicine and Health, University of Manchester, Manchester M13 9PL, UK; 7 Dxcover Ltd., Glasgow G33 1AP, UK; 8 WestCHEM, Department of Pure and Applied Chemistry, University of Strathclyde, Glasgow G1 1XL, UK; 9 Danish Comprehensive Cancer Center Brain Tumor Centre, 2100 Copenhagen, Denmark; 10 Department of Oncology, University Hospital of Copenhagen, 2100 Copenhagen, Denmark; 11 School of Health & Life Sciences, Teesside University, Middlesbrough TS1 3BX, UK; 12 National Horizons Centre, Teesside University, Darlington DL1 1HG, UK; 13 School of Medicine, Faculty of Health Sciences, Aristotle University of Thessaloniki, 541 24 Thessaloniki, Greece; 14 Department of Neurosurgery, Kings College Hospital NHS Foundation Trust, London SE5 9RS, UK; 15 Department of Neurosurgery, Beijing Tiantan Hospital, Capital Medical University, Beijing 100070, China; 16 Department of Neurosurgery, University Hospital of Copenhagen, 2100 Copenhagen, Denmark; 17 School of Medicine, University of Central Lancashire, Preston PR1 7BH, UK; 18 MRC Clinical Trials Unit, University College London, London WC1V 6LJ, UK; 19 Department of Neurosurgery, University Hospitals Sussex NHS Foundation Trust, Brighton BN2 5BE, UK

**Keywords:** liquid biopsy, extracellular vesicle, glioma, glioblastoma, multi-omics, spectroscopy, blood, machine-learning, biomarker, brain tumor

## Abstract

Plasma small extracellular vesicles (sEVs) are a promising liquid biopsy tool. This study aims to delineate and validate a multimodal plasma sEV biomarker signature for glioma. We use size exclusion chromatography to separate sEVs from plasma (1 mL) and a combination of multi-spectral (Fourier transform infrared/Raman) and orthogonal multi-omics (proteomic/microRNA) approaches on 206 plasma samples (159 individuals) across three independent cohorts. We identify distinct glioma sEV biomolecular profiles, including differences in sEV protein/nucleic acid composition, and consistent alterations in 45 proteins and 20 microRNAs. Machine learning models derived from training cohort data achieve high diagnostic performance (areas under the curve [AUCs] 0.931–0.971), while external validation across independent cohorts confirms the signature’s diagnostic potential, with 100% accuracy for the proteomic and multimodal signatures in the longitudinal cohort. Our findings, generated through a rigorous multi-cohort and multi-algorithmic framework, establish the potential of plasma sEV signatures as a clinically relevant diagnostic liquid biopsy approach for glioma.

## Highlights

• Glioma patient sEVs differ markedly compared to controls and other brain tumors
• Multi-cohort validation of sEVs results in a highly accurate biomarker signature
• sEVs have potential as a liquid biopsy to support in the diagnosis of glioma

## Graphical abstract

**Figure:**
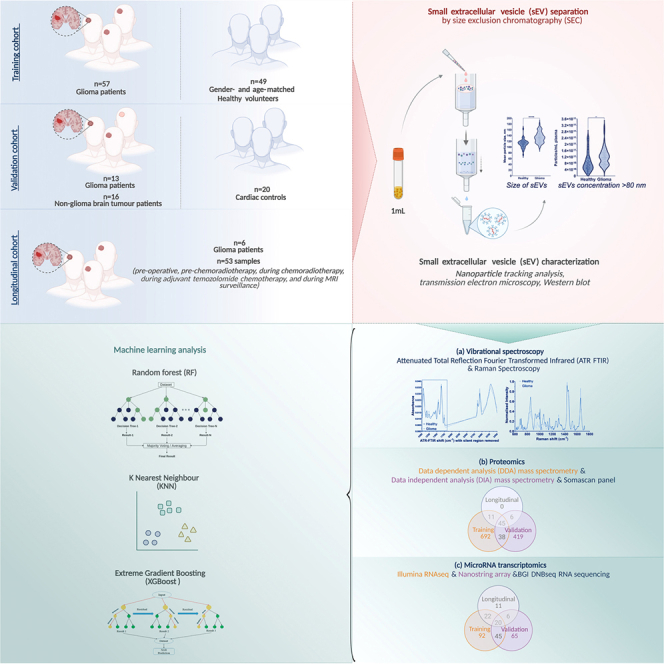

## Teaser

Robinson et al. identify distinct plasma small extracellular vesicle characteristics, vibrational spectral signatures, and multi-omics analysis (protein/microRNA) for glioma patients. By using machine learning techniques, they develop a highly accurate biomarker signature that discriminates glioma patients from healthy volunteers, subsequently validating this approach through orthogonal omics analysis in two external cohorts.

## Introduction

Gliomas, the most common primary brain tumors, constitute a leading cause of absolute years of life lost,^1^ reflecting their profound societal burden. Due to their intracranial location, diagnosis requires neurosurgical intervention, which also often serves a therapeutic purpose, thereby limiting the scope for pre-operative treatment strategies. Glioma classification is fundamentally guided by the mutational status of the isocitrate dehydrogenase (IDH) enzyme, distinguishing IDH wild-type (IDH^wt^) glioblastoma from IDH mutant (IDH^mut^) glioma subtypes,^2^ with diverging clinical management^3^ and prognoses (median overall survival of approximately 9 versus 21–207 months, respectively^4^). Consequently, the development of minimally invasive liquid biopsy platforms capable of molecular glioma subtyping represents a critical unmet need in neuro-oncology.^5^ However, conventional liquid biopsy analytes, including circulating tumor cells (CTCs) or circulating tumor DNA (ctDNA), are often undetectable in the peripheral circulation, limiting their utility.^6^^,^^7^

Analysis of plasma-derived small extracellular vesicles (sEVs) has emerged as a promising biomarker discovery approach. sEVs are lipid-encapsulated nanoparticles containing bioactive molecular cargo (proteins, RNA, etc.), reflective of their cellular origins.^8^ We have previously demonstrated sEV proteomics potential for distinguishing IDH^wt^ glioblastoma from healthy controls,^9^ with complementary reports that sEV RNA signatures can differentiate glioma subtypes,^10^ and sEV concentrations correlate with tumor burden.^11^ Additionally, emerging evidence suggests that vibrational spectroscopy can identify spectral “fingerprint” characteristics of glioma in tumor tissue^12^^,^^13^ and serum.^14^

**Figure 1:**
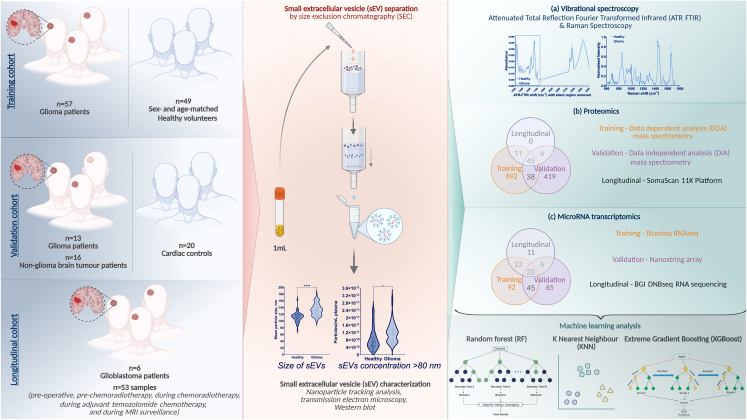
Flow diagram describing the study overview and workflow

Here, we aimed to define and externally validate a comprehensive biomarker signature for glioma detection and subtyping. This was achieved through a multimodal interrogation (incorporating spectral and orthogonal proteomic and microRNA analyses) of sEVs separated from 1 mL plasma samples across three independent cohorts (*n* = 206 samples from 159 individuals) (Figure 1).

Our analysis revealed significant alterations in the plasma sEV landscape in glioma patients, including increased vesicle size and concentration. We identified marked differences in sEV composition between glioma patients and controls using both attenuated total reflection Fourier transform infrared (ATR-FTIR) and Raman spectrometry, alongside consistent proteomic and microRNA alterations using orthogonal approaches in independent cohorts. Using machine-learning algorithms, we developed and externally validated diagnostic models that effectively distinguished glioma patients from controls (training cohort areas under the curve [AUCs] 0.931–0.971; internal validation accuracy: 85%–95%; external validation accuracy: 40%–100%). Exploratory analysis also revealed sEVs-based differences between glioma subtypes and other brain tumor entities.

Overall, we demonstrate the technical feasibility and clinical potential of pre-operative plasma sEV analysis as a transformative tool for the diagnosis and molecular subtyping of glioma, which could ultimately guide personalized therapeutic decision-making.

## Results

### Plasma sample cohort characteristics

To evaluate the diagnostic potential of plasma sEVs for glioma, three independent cohorts of plasma samples were obtained and analyzed (Figure 1 and Table S1).

The training cohort comprised pre-operative plasma from glioma patients (*n* = 56) from the 100K Genome Project alongside gender-/age-matched healthy volunteers (*n* = 48). The glioma group was predominantly male (63%) with a mean age of 48.1 years compared to 46.3 years for the healthy volunteers. According to the World Health Organization (WHO) CNS 5 criteria,^2^ the cohort included 33 IDH^wt^ glioblastomas (59%) and 20 IDH^mut^ gliomas (36%) (Tables S1 and S2).

The validation cohort included pre-operative plasma from WHO-CNS-5-defined glioma patients (*n* = 13; 9 IDH^wt^ glioblastoma and 4 IDH^mut^ glioma) and patients with other brain tumors (*n* = 16; eight meningioma, seven brain metastases, and one lymphoma) from the Dunedin Brain Tumor database, alongside control plasma from cardiac disease patients (*n* = 20) from the HeartOtago database (Table S1).

The longitudinal cohort consisted of 53 plasma samples from six WHO-CNS-5-defined IDH^wt^ glioblastoma patients enrolled in the Neurogenome study,^15^ collected at defined time points: pre-operative (*n* = 5), pre-chemoradiotherapy (*n* = 6), during chemoradiotherapy (2–3 samples, *n* = 6), during adjuvant temozolomide chemotherapy (2–6 samples, *n* = 6), and during MRI surveillance (2 samples, *n* = 2). Of these samples, four were taken after MRI-defined progression (Table S1).

### Plasma sEV profiling differentiates glioma patients from healthy volunteers

sEVs were successfully separated from all 1 mL plasma samples using size exclusion chromatography^16^ (Figure S1).

**Figure 2:**
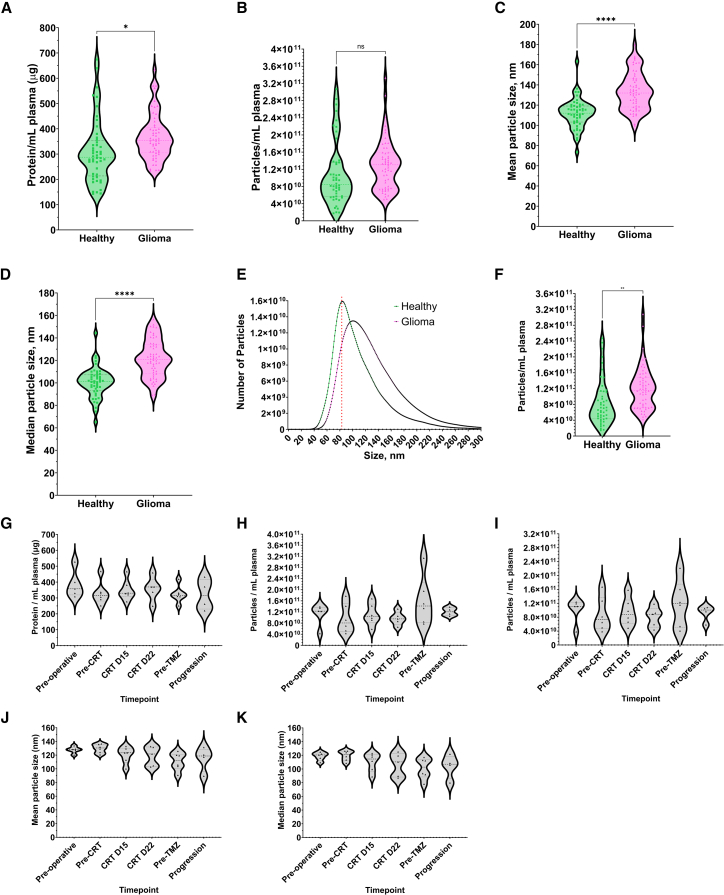
Sample characterization of plasma sEVs according to the minimal information for studies of extracellular vesicles guidelines differentiates glioma patients from gender- and age-matched healthy volunteers within the training cohort, with similar findings among the glioblastoma patients within the longitudinal cohort (A) Within the training cohort (*n* = 104), (A) protein levels are increased in glioma patient samples as quantified by microBCA. (B–D) Nanoparticle tracking analysis identified no difference in total particle concentration but identified increases in (C) mean and (D) median particle size. (E) There is a clear increase in particle size across the whole size distribution for glioma patients as assessed by nanoparticle tracking analysis, while the size cutoff for alternative size exclusion chromatography columns (80 nm) is highlighted by a dotted red line. (F–H) Using a particle size cutoff of 80 nm, there is an increase in the number of particles from glioma patients’ plasma compared to healthy volunteers’ plasma. Within the longitudinal cohort (*n* = 6 [*n* = 53 samples]), (G) similar elevations in the protein levels were identified in the pre-surgical glioblastoma patient samples. Nanoparticle tracking analysis identified (H) similar total particle concentrations with (I) elevated >80 nm particle concentrations, which seemed to track patient disease state. Nanoparticle tracking analysis also demonstrated elevated (J) mean and (K) median particle size, which also seemed to track patient disease state. Data presented as violin plots and difference assessed using Welch’s unpaired *t* test; ns *p* ≥ 0.05; ∗*p* < 0.05, ∗∗*p* < 0.01, ∗∗∗*p* < 0.001, ∗∗∗∗*p* < 0.0001.

Training cohort sEV protein concentrations were elevated with glioma (368.0 vs. 310.8 μg/mL; *p* = 0.0104; Figure 2A). While total particle yield was similar (1.30 × 10^11^ vs. 1.09 × 10^11^ particles/mL; *p* = 0.5645; Figure 2B), both mean (133.6 vs. 111.9 nm, *p* < 0.0001; Figure 2C) and median (121.6 vs. 100.5 nm, *p* < 0.0001; Figure 2D) particle sizes were significantly increased in glioma patients, with a shift toward larger sEV subpopulations (Figure 2E). Using an 80 nm threshold, aligned with other size exclusion chromatography column exclusion limits, we observed an increase in particle yield (1.20 × 10^11^ vs. 8.51 × 10^10^ particles/mL plasma, *p* = 0.0024) for glioma patients (Figure 2F).

The longitudinal cohort’s pre-operative samples demonstrated similarly elevated sEV protein values (384 μg/mL; Figure 2G), unchanged total particle yield (1.12 × 10^11^ particles/mL; Figure 2H) but increased yield of >80 nm particles (1.01 × 10^11^ particles/mL; Figure 2I), mean size (128 nm; Figure 2J), and median size (119 nm; Figure 2K) for IDH^wt^ glioblastoma patients. Interestingly, longitudinal assessment of >80 nm particle yield plus particle size dynamics suggested an association with tumor status (Figures 2I–2K).

### Vibrational spectroscopy analysis of sEVs identifies glioma patients

To investigate the sEV sample composition, alongside the potential to discriminate glioma patients versus healthy volunteers, ATR-FTIR spectroscopy was employed using the Dxcover platform.

**Figure 3:**
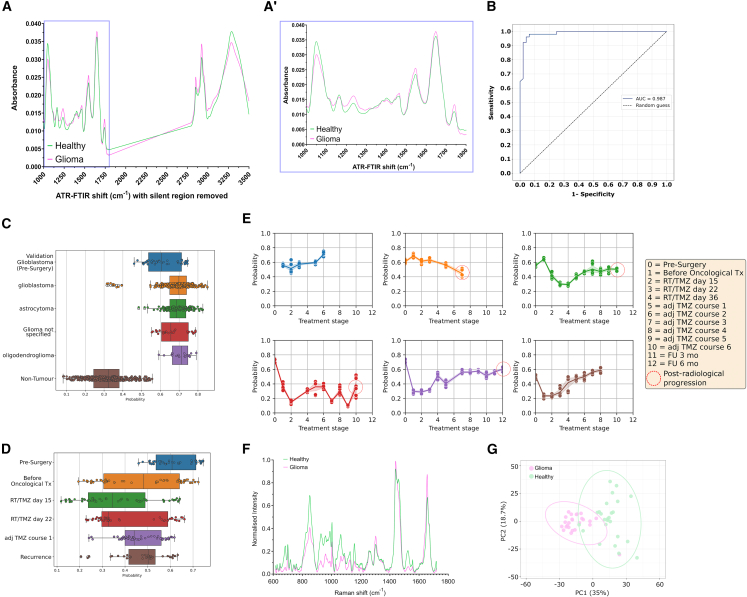
Spectroscopic analysis of the separated sEV sample identifies clear differences in chemical composition between glioma patients’ samples and healthy volunteers’ samples (A) In the training cohort (*n* = 99), (A) ATR-FTIR spectra of the sEVs from glioma patients and healthy volunteers have distinct patterns with clear differences in several peaks across the whole spectrum (1,000–3,500 cm^−1^ wavenumbers with the silent region of 1,800–2,800 cm^−1^ removed) and (A′) the fingerprint region (1,000–1,800 cm^−1^ wavenumbers). (B and C) Receiver operating characteristic analysis for the top performing model (partial least-squares regression model) in the training cohort. In the longitudinal cohort (*n* = 6 [*n* = 53 samples]), (C) the best performing model from the training cohort generates prediction probabilities most in keeping with a glioma diagnosis. (D) Longitudinal evaluation of the mean prediction probability demonstrates a reduction compared to the pre-operative sample values with no clear subsequent trajectory. (E) Longitudinal evaluation of the prediction probabilities for the individual patients demonstrates no consistent trend in response to treatment. (F) Raman spectra (normalized intensities) of the analyzed sEVs from glioma patients (*n* = 25) and healthy volunteers (*n* = 25) have distinct patterns with clear differences in several peaks. (G) PCA plot shows the grouping of healthy volunteer sEV and glioma sEV samples based on their Raman spectra. All the patients (normalized data) were plotted for the two largest PCs (PC1 and PC2) and clustered as a function of their group biomolecular variations.

Training cohort sample analysis revealed distinct sEV spectral profiles across the fingerprint (1,800–1,000 cm^−1^) and higher (3,500–2,800 cm^−1^) wavenumber regions (Figure 3A). Inferences regarding sample biomolecular composition can be made from alterations in peaks based on previously published data.^17^ Recursive feature elimination (RFE) identified 10 most discriminative peaks at 1,133 cm^−1^/1,134 cm^−1^ (nucleic acids); 1,240 cm^−1^ (nucleic acids and lipid/protein C-O stretching); 1,263 cm^−1^/1,265 cm^−1^/1,285 cm^−1^ (Amide III); 1,453 cm^−1^ (proteins CH_3_ bending); 1,797 cm^−1^ (lipids C=C stretching); 3,016 cm^−1^ (CH deformations in lipids); and 3,500 cm^−1^ (O-H stretching) (Table S3).

A partial least-squares regression model identified a biomarker signature that differentiated glioma patients from controls with high accuracy (AUC 0.987; Figure 3B). Application of this model to longitudinal cohort samples confirmed its generalizability, albeit with slightly lower confidence scores (Figure 3C). Additionally, the longitudinal assessment of the ATR-FTIR spectra revealed that the diagnostic probability was highest pre-operatively, declined with treatment, and increased at recurrence (Figure 3D). Despite inter-patient variability, individual patient trajectories showed a marked reduction in diagnostic probability during treatment (*n* = 4), followed by an increase at recurrence (*n* = 3) (Figure 3E).

Additionally, in a training cohort subset (*n* = 25 glioma, *n* = 25 control), Raman spectroscopy corroborated these findings, showing distinct spectral profiles (Figure 3F), with good separation via principal-component analysis (PCA) (Figure 3G). Subsequently, RFE identified 10 key Raman peaks (Table S3). Using published data,^18^ these peaks were assigned to molecular characteristics that demonstrate differences in the nucleic acid (677 cm^−1^/682 cm^−1^/685 cm^−1^/789 cm^−1^/913 cm^−1^) and protein (Amide II: 1,507 cm^−1^/1,529 cm^−1^; Amide I: 1,642 cm^−1^/1,643 cm^−1^/1,645 cm^−1^) composition of sEVs.

Collectively, these complementary vibrational spectroscopy data demonstrate altered protein and nucleic acid (including RNA) composition in plasma sEVs from glioma patients compared to healthy volunteers.

### Proteomics profiling of sEVs enables glioma discrimination

**Figure 4:**
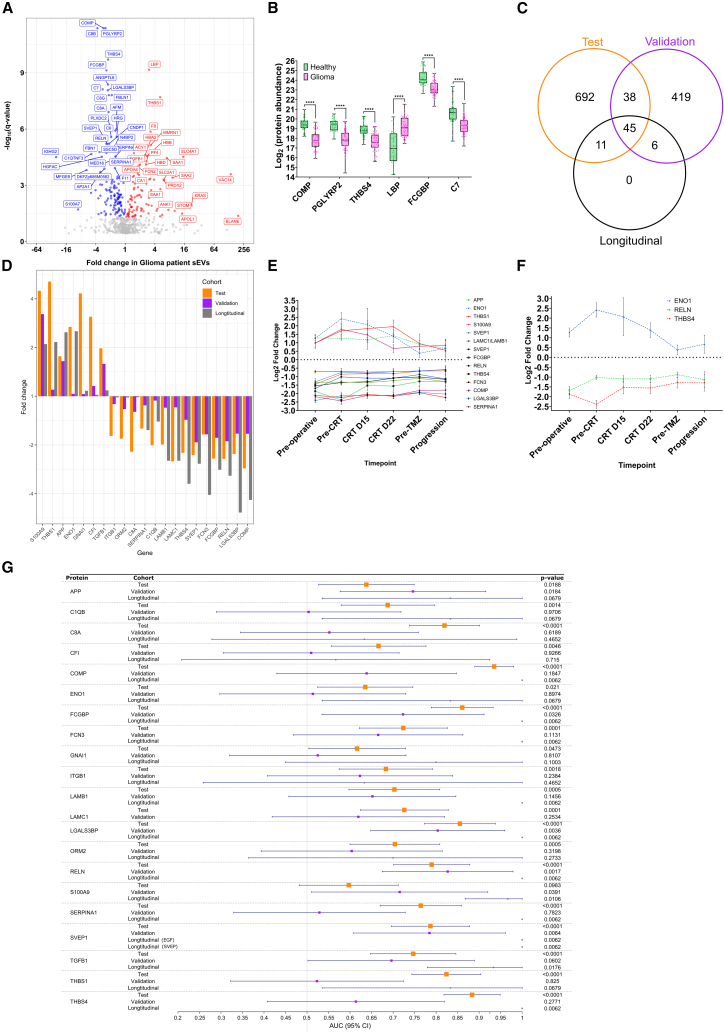
Proteomic characterization of circulating sEVs by orthogonal techniques demonstrates coherent protein changes across independent cohorts containing glioma patients and controls (A) In the training cohort (*n* = 99), (A) volcano plot displays the relationship between fold change and statistical significance of the identified differentially abundant proteins with a significance threshold of *q* < 0.05. (B) Protein abundance profiles of selected protein biomarkers (fold change ≥2 and *q* < 0.01). (C) Venn diagram demonstrating the overlap in identified protein abundance changes between the training (*n* = 99) and validation (*n* = 49) cohorts. (D–F) Bar chart demonstrating the consistent fold change of the concordant proteins (*n* = 22) between the three cohorts (training: *n* = 99, validation: *n* = 49, and longitudinal: *n* = 6 [*n* = 53 samples]). Longitudinal assessment of the most differentially abundant concordant proteins (fold change >±1.5 in the pre-treatment samples of the longitudinal cohort, *n* = 14) demonstrates persistent alteration during treatment (E), while for select proteins (*n* = 3), a degree of normalization occurs during treatment with minimal change at recurrence (F). (G) Forest plot comparing the area under the receiver operating characteristic curve for the top protein biomarkers. The longitudinal analysis assessed levels of the LAMB1/LAMC1 complex and two SVEP1 epitopes. Statistical analyses were performed using Welch’s *t* test; ns *p* ≥ 0.05; ∗*p* < 0.05, ∗∗*p* < 0.01, ∗∗∗*p* < 0.001, ∗∗∗∗*p* < 0.0001. See also Tables S5, S6, S7, and S8.

To investigate plasma sEV protein composition, unbiased, label-free mass spectrometry was performed on training cohort samples, identifying 887 protein species, with 775 proteins common to glioma patients and health volunteers (Table S5). Comparative analysis revealed 305 differentially abundant proteins (*q* < 0.05; Figure 4A; Table S5). Applying more stringent criteria (*q* < 0.01, fold change >±1.5, unique peptides >1) yielded 78 candidate biomarker proteins (Table S5), while the top six proteins (*p* < 0.001) demonstrated robust differential abundance (Figure 4B). Functional analysis of the 305 differentially abundant proteins revealed a highly interactive network (*p* < 1.0 × 10^16^; Figure S2A), and WikiPathways analysis implicated dysregulation of complement and coagulation cascades and enrichment in cholesterol-metabolism-associated proteins (Figure S2B), processes previously associated with glioma.

We validated these findings using an alternative mass spectrometry approach for Validation cohort samples. This reliably identified (present in ≥67% of samples) 502 proteins (Table S6), following filtering of common contaminants (keratins/immunoglobulins/apolipoproteins/albumin). Cross-referencing identified 140 proteins (>1 unique peptide) detected in both cohorts, with 83 exhibiting concordant abundance changes (Figure 4C; Table S7). Within this set, 51 proteins were differentially abundant (*q* < 0.05) and 35 also had a fold change >±1.5 in the training cohort (Table S7). Interestingly, 20 proteins that were differentially abundant (*q* < 0.05) and with a fold change >±1.5 showed discordant abundance patterns between cohorts (Table S7). STRING pathway analysis revealed a highly interconnected network for the 83 concordant proteins (*p* < 1.0 × 10^−16^; Figure S2C), compared to a weaker network for the 20 discordant proteins (*p* = 0.0101; Figure S2E). WikiPathways analysis of concordant proteins indicated inhibition of pathways related to complement activation and cell adhesion, alongside increases in reactive oxygen species regulation and pro-inflammatory pathways (Figure S2D). Discordant proteins were only associated with inhibition of the membrane attack complex (Figure S2F).

We further refined these findings by performing targeted (non-mass-spectrometry) proteomics. We successfully identified the 83 concordant proteins, plus 17 additional candidates identified across both previous cohorts (Table S4), in longitudinal cohort samples alongside matched controls (*n* = 6). Across all three cohorts, 45 proteins had consistent abundance changes (Figure 4C; Table S8), while 22 were highly differentially abundant (*q* < 0.05, fold change >±1.5) in the training cohort (Figure 4D; Table S8). Of these 22 highly concordant proteins, 14 had a fold change >±1.5 in the longitudinal cohort (Figure 4D) and each remained differentially abundant compared to the matched controls at all time points (Figure 4E). Although limited by sample size, a subgroup of these proteins may be associated with treatment response, with a degree of protein abundance normalization following treatment, albeit with minimal change at recurrence (Figure 4F). Individually, the 22 highly concordant proteins (training *q* < 0.05, fold change >±1.5) showed variable discriminatory power in receiver operating characteristic (ROC) analysis (training cohort AUC 0.597–0.935; Figure 4G). However, the top four proteins demonstrated good to excellent diagnostic accuracy (training cohort AUC: 0.787–0.861, *p* < 0.05 across cohorts) (Figures 4G and S3A–S3C).

### sEVs microRNA transcriptomics enables glioma discrimination

**Figure 5:**
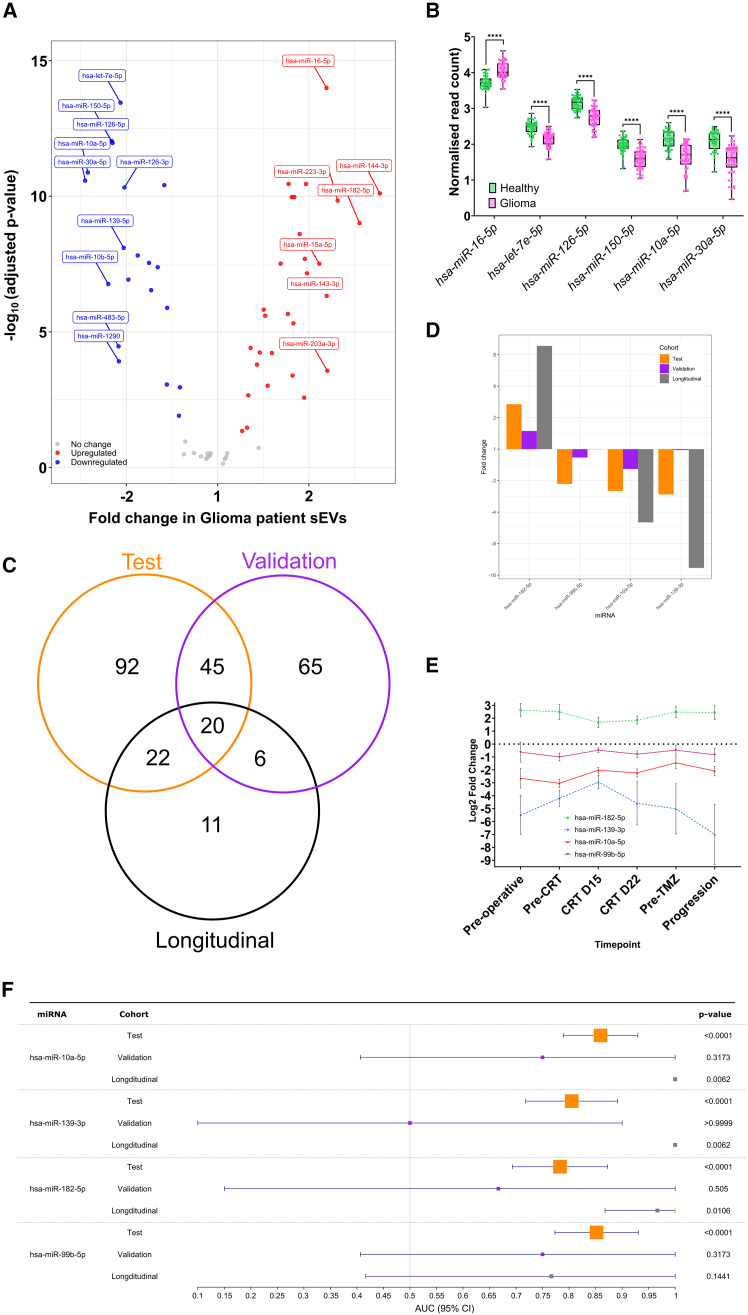
Orthogonal transcriptomic characterization of circulating sEV microRNA identifies consistent alterations between glioma patients and controls across independent cohorts (A) In the training cohort (*n* = 96), (A) volcano plot displays the relationship between fold change and statistical analysis of the identified differentially expressed microRNA with a threshold of adjusted *p* < 0.05. The colored microRNA represents those with *p* ≤ 0.01 and fold change >±2. (B) MicroRNA expression profiles of selected biomarkers (fold change ≥±2 and adjusted *p* < 0.01). (C) Venn diagram demonstrating the overlap in identified miRNA expression alterations between the training (*n* = 96) and validation (*n* = 12) cohorts. (D) Bar chart demonstrating the consistent fold change of the concordant microRNA (*n* = 20) between the three cohorts (training: *n* = 96, validation: *n* = 12, and longitudinal: *n* = 6 [*n* = 53 samples]). (E) Longitudinal assessment of the most differentially expressed microRNA (*n* = 4) highlights persistent differential expression throughout treatment, while for select microRNA (*n* = 3) there is a suggestion that their values may track tumor status. (F) Forest plot comparing the area under the receiver operating characteristic curve for the top microRNA biomarkers. Statistical analyses were performed using Welch’s *t* test; ns *p* ≥ 0.05; ∗*p* < 0.05, ∗∗*p* < 0.01, ∗∗∗*p* < 0.001, ∗∗∗∗*p* < 0.0001. See also Tables S9, S10, S11, and S12.

To complement our proteomic profiling, we performed next-generation-sequencing-based microRNA transcriptomics on training cohort sEVs. This consistently detected 152 microRNAs (Table S9), of which 77 were differentially expressed (adjusted *p* < 0.05) and 17 also exhibited a fold change >±2 (Figure 5A; Table S9). The six top candidate microRNAs showed robust differential expression (*p* < 0.001 for each; Figure 5B). KEGG pathways analysis associated these microRNAs with “pathways in cancer,” “microRNAs in cancer,” and “glioma” as three of the most frequently impacted pathways. Additionally, “FOXO signaling,” “PI3K/AKT signaling pathway,” “focal adhesion,” and “HIF-1” signaling pathways were identified in keeping with known pathways altered in glioma (Figure S4A).

We validated these findings by comparing sequencing data (Illumina; training cohort) with microarray results (NanoString; validation cohort). Among 131 microRNAs consistently detected in both cohorts, 65 exhibited a concordant expression pattern in glioma samples (Figure 5C; Table S10). This included 12 miRNAs that were highly differentially expressed (training adjusted *p* < 0.05, fold change >±2) (Table S10). In contrast, 16 highly differentially expressed microRNAs (adjusted *p* < 0.05, fold change >±2) showed discordant patterns between cohorts (Table S10). KEGG pathway analysis of the 12 concordant microRNAs (training cohort adjusted *p* < 0.05, fold change >±2) confirmed enrichment in “pathways in cancer,” “microRNAs in cancer,” “glioma,” and “FoxO signaling pathways” (Figure S4B). Discordant microRNAs (training cohort adjusted *p* < 0.05 and fold change >±2) were associated with vascular (“AGE-RAGE signaling pathway in diabetic complications” and “fluid shear stress and atherosclerosis”) and infection-related (“hepatitis B” and “hepatitis C″) pathways (Figure S4C).

Further validation was performed using an alternate microRNA sequencing method, DNBSeq, on longitudinal cohort samples alongside matched controls (*n* = 6). From the 59 microRNAs consistently identified across control and pre-operative samples (Table S11), 20 microRNAs had a concordant differential expression pattern across all three cohorts (Figure 5C; Table S12). Of these, 14 microRNAs were differentially expressed (adjusted *p* < 0.05) in the training cohort, with four microRNAs also having a fold change >±2 in the training cohort (Figure 5D; Table S12). Longitudinal assessment revealed stable differential expression for three of these microRNAs, with trends suggesting normalization during treatment and re-emergence at recurrence (Figure 5E). ROC curve analysis identified that all four microRNA could identify glioma patients across the three cohorts (training AUCs: 0.783–0.859) (Figures 5F and S3D–S3F).

### Machine learning defines biomarker signatures with clear diagnostic potential

To define a glioma biomarker signature, we implemented a comparative machine learning framework evaluating three separate machine learning algorithms (Random Forest models [RF], k-Nearest Neighbor [kNN], and Extreme Gradient Boosting [XGBoost] algorithms).

**Figure 6:**
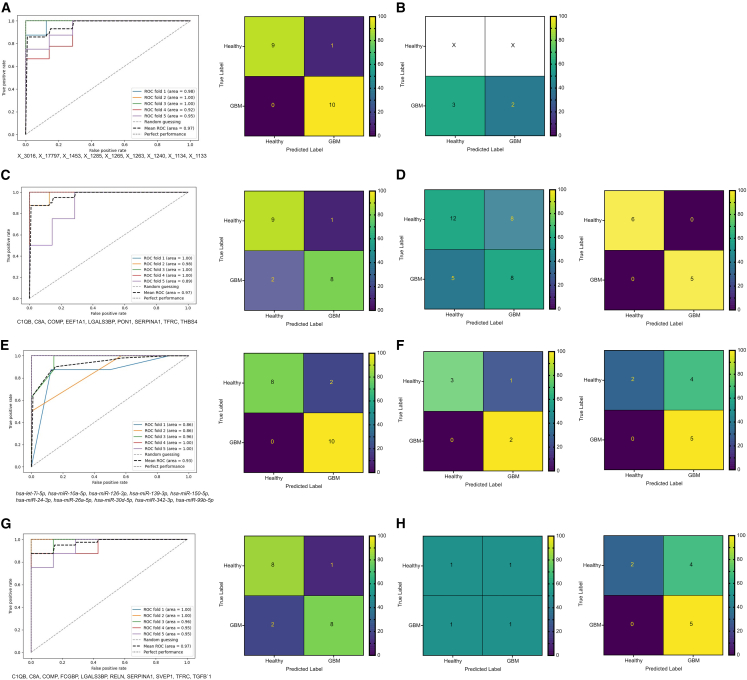
Performance evaluation of the developed machine learning models based on plasma sEV characteristics and multimodal analysis demonstrates excellent accuracy in glioma classification The ensemble receiver operating characteristic curve for the 5-fold cross-validation performance of the generated model in terms of the area under the curve across each of the 5-folds to provide insights into the robustness of each model and confusion matrix of the internal validation subset of the training cohort for the (A) sEV ATR-FTIR spectra (XGBoost, *n* = 51 glioma, *n* = 48 control), (C) sEV protein (XGBoost, *n* = 51 glioma, *n* = 47 control), (E) sEV microRNA (kNN, *n* = 49 glioma, *n* = 47 control), and (G) combined sEV protein and microRNA (RF, *n* = 49 glioma, *n* = 46 control). With the corresponding confusion matrices when assessing the identified signatures within the independent cohorts, for the (B) sEV ATR-FTIR spectra (longitudinal: *n* = 5 glioblastoma), (D) sEV protein (validation: *n* = 13 glioma, *n* = 20 control; longitudinal: *n* = 5 glioblastoma, *n* = 6 control), (F) sEV microRNA (validation: *n* = 2 glioma, *n* = 4 control; longitudinal: *n* = 5 glioblastoma, *n* = 6 control), and (H) combined (validation: *n* = 2 glioma, *n* = 2 control; longitudinal: *n* = 5 glioblastoma, *n* = 6 control). See also Tables S13 and S14.

For ATR-FTIR spectroscopy, the XGBoost algorithm generated the optimal model (Table S13), leading to a nine-feature signature (Figure S5A) with a training cohort AUC of 0.966 (Figure 6A), without evidence of overfitting (Figure S6A). However, validation in the longitudinal cohort (*n* = 5) yielded lower accuracy (40%; Figure 6B; Table S14), suggesting batch differences between the cohorts despite consistent processing.

For the proteomic, microRNA, and combined *Z* scored datasets, PCA demonstrated no clear batch effects between the cohorts (Figures S5B–S5D).

Subsequently, the XGBoost algorithm generated the optimal protein model (Table S13), resulting in a nine-feature signature (Figure S5E) with a training cohort AUC of 0.971 (Figure 6C), without evidence of overfitting (Figure S6B). Evaluating this signature in the two external cohorts resulted in a reduced accuracy of 61% in the validation cohort (*n* = 33) but perfect accuracy (100%) in the longitudinal cohort (*n* = 11) (Figure 6D; Table S14).

For microRNA, the kNN algorithm generated the optimal model (Table S13), resulting in a 10-feature signature (Figure S5F) with a training cohort AUC of 0.931 (Figure 6E), without evidence of overfitting (Figure S6C). Evaluating this signature in the external cohorts resulted in an accuracy of 83% (100% sensitivity) in the validation cohort (*n* = 6) and 64% accuracy (100% sensitivity) in the longitudinal cohort (*n* = 11) (Figure 6F; Table S14).

Subsequently, a multimodal (protein/microRNA) model was generated using the RF algorithm (Table S13), resulting in a 10-feature protein-only signature (Figure S5G), with a training cohort AUC of 0.971 (Figure 6G), without evidence of overfitting (Figure S6D). This signature had perfect accuracy in differentiating glioma patients from controls in the longitudinal cohort (*n* = 11) but only 50% accuracy in the validation cohort subgroup with protein and microRNA data (*n* = 4) (Figure 6H; Table S14).

### Multimodal plasma sEV signature analysis distinguishes glioma subtypes and non-glioma tumors

Given their different prognosis and treatment, we performed an exploratory analysis to determine if plasma sEV profiling could differentiate IDH^wt^ glioblastoma patients (*n* = 33) from IDH^mut^ glioma patients (*n* = 20) in the training cohort and between glioma (*n* = 13) and non-glioma brain tumors (*n* = 16, Table S1).

**Figure 7:**
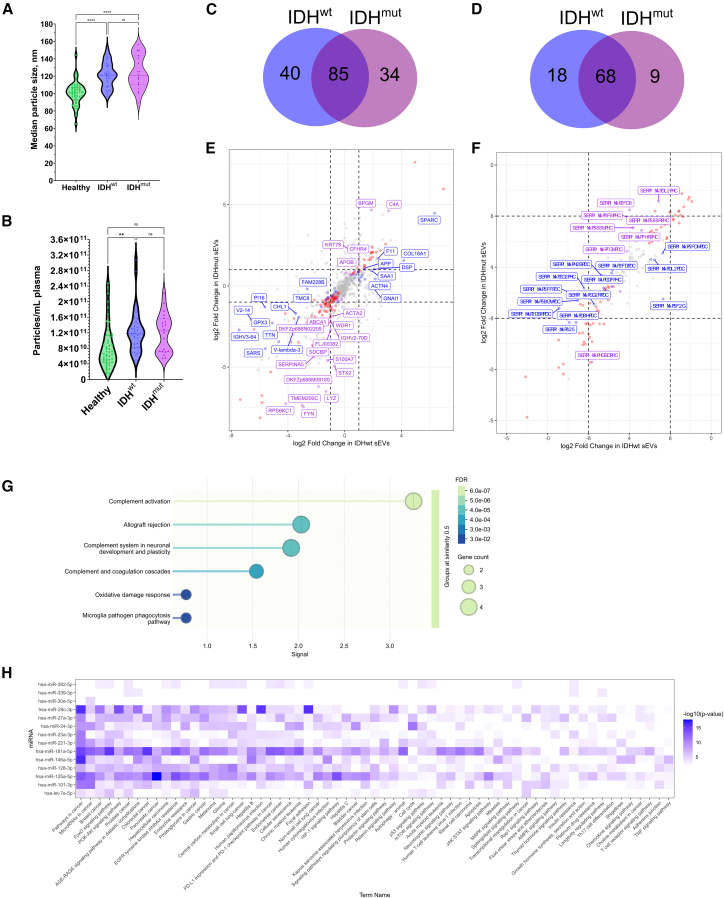
Comparison of sEV characteristics, -omics, and spectral features can differentiate IDH^wt^ glioblastoma patients (*n* = 33) from IDH^mut^ glioma patients (*n* = 20) and glioma patients (*n* = 13) from non-glioma brain tumor patients (*n* = 16) (A and B) Nanoparticle tracking analysis comparing (A) median particle size and (B) particles >80 nm concentration does not clearly differentiate IDH^wt^ glioblastoma patients from IDH^mut^ glioma patients and healthy volunteers. (C and D) Venn diagrams demonstrating the overlap in (C) differentially abundant proteins and (D) differentially expressed microRNA for IDH^wt^ glioblastoma patients’ and IDH^mut^ glioma patients’ sEVs compared to healthy volunteer sEVs. (E and F) Scatterplots displaying the relationship between the fold change of the (E) differentially abundant proteins and (F) differentially expressed microRNA for IDH^wt^ glioblastoma patients’ and IDH^mut^ glioma patients’ sEVs compared to healthy volunteer sEVs. The dashed lines represent fold change >±2. (G) WikiPathways enrichment analysis of the proteins with a reversed pattern of differential abundance between the non-glioma brain tumor and control samples. (H) KEGG pathway analysis of the microRNA with a reversed pattern of differential expression between the non-glioma brain tumor samples and controls. Data presented as violin plots and difference assessed using one-way ANOVA with post-hoc Tukey; ns *p* ≥ 0.05; ∗*p* < 0.05, ∗∗*p* < 0.01, ∗∗∗*p* < 0.001, ∗∗∗∗*p* < 0.0001. See also Tables S8 and S12.

Analysis of sEV characteristics identified similar particle sizes and concentrations of particles >80 nm between glioblastoma and IDHmut gliomas, although both subtypes exhibited increased values compared to controls (Figures 7A and 7B). However, when compared to controls, 125 and 119 proteins were differentially abundant in glioblastoma and IDHmut gliomas, respectively, with an overlap of 85 proteins (Figures 7C–7E). Similarly, 86 and 77 microRNAs were differentially expressed in IDH^wt^ glioblastoma patients and IDH^mut^ glioma patients, respectively, with 68 differentially expressed microRNA common to both subtypes (Figures 7D–7F).

Comparing the proteomic and microRNA sEV content between glioma and non-glioma brain tumor patients in the validation cohort demonstrated 14 of 83 concordant proteins with a reversed differential abundance pattern between non-glioma brain tumor samples and controls relative to glioma versus control samples (Table S8). These 14 proteins were associated with upregulation of complement activation and the oxidative damage response (Figure 7G). Whereas 26 of 65 concordant microRNAs had reversed patterns of differential expression between non-glioma brain tumors and control samples relative to glioma versus control samples (Table S12). Analysis of the KEGG pathways associated with these microRNAs identified “pathways in cancer,” “breast cancer,” and “prostate cancer” as three of the most frequently impacted pathways in keeping with the tumor characteristics of the non-glioma brain tumor samples analyzed (Figure 7H).

## Discussion

The development of a minimally invasive, liquid biopsy platform for brain tumors holds transformative potential across the clinical continuum, including faster diagnosis, treatment monitoring, and early recurrence detection.^19^ Several different techniques are under investigation, namely circulating tumor DNA (ctDNA),^20^ circulating tumor cells (CTC),^21^ and tumor-educated platelets^22^; however, their clinical translation is hindered by several challenges, including technical complexity, low analytical sensitivity, large sample volume requirements, and/or high costs.

sEVs have emerged as an alternative approach due to their unique characteristics. They demonstrate prolonged stability within the circulation,^23^ allowing processing ≤8 h after collection with appropriate processing^24^ facilitating their translation to clinical settings. Further, their increased blood concentrations, compared to ctDNA/CTC,^23^ provides excess yield from ≤1 mL plasma,^16^ and sEVs seem to be enriched for potential biomarkers compared to unprocessed blood components.^25^ sEV separation and analysis techniques continue to advance, including exciting developments in microfluidics and single-EV analysis.^26^ For example, using high-resolution density gradient fractionation to separate EVs, Rai et al.^27^ performed a highly specific quantitative proteome and lipidome analysis to differentiate EV and non-EV particles in plasma. However, these techniques are technically challenging, often with significant infrastructure costs, and are therefore not feasible in most clinical settings.^28^

In this study, we employed a standardized size exclusion chromatography protocol to separate sEVs from 1 mL plasma.^16^ We identified clear and reproducible differences in plasma sEVs from glioma patients versus healthy volunteers across three independent cohorts. Consistent with previous reports,^11^^,^^29^^,^^30^ we observed elevated plasma sEV concentrations in glioma patients, particularly the >80 nm subpopulation, with trends suggesting correlation with clinical status. sEV concentration has previously been associated with the extent of FLAIR hyperintensity on MRI and extent of resection,^29^ shown to reduce after chemoradiotherapy,^30^ and increase upon recurrence,^11^^,^^29^ highlighting sEVs potential as a monitoring biomarker. Additionally, despite discordant findings in the literature,^11^^,^^29^^,^^30^ we identified increased particle size for glioma patients, with similar increased particle size across independent cohorts, alongside a longitudinal pattern that tracks clinical status.

Moving beyond prior single-modality sEVs studies in glioma,^10^^,^^11^^,^^29^^,^^30^^,^^31^^,^^32^^,^^33^ we conducted a comprehensive multimodal analysis using vibrational spectroscopy (ATR-FTIR and Raman), proteomics (mass spectrometry and non-mass spectrometry techniques), and microRNA profiling (next-generation-sequencing-based microRNA transcriptomics and microRNA array analysis).

Vibrational spectroscopy analysis is increasingly being investigated in neuro-oncology, including Raman spectroscopy as an intra-operative diagnostic^13^ and ATR-FTIR as a triage liquid biopsy on serum.^14^ Encouragingly, we identified distinct plasma sEV spectral signatures for glioma patients and controls. Through comparing the assignment of ATR-FTIR^17^ and Raman^18^ spectroscopy peaks, we demonstrate clear differences in protein and nucleic acid composition. Employing a label-free Raman spectroscopy technique, we generated sufficient signal from a 10 μL sEV sample. Alternatively, surface-enhanced Raman spectroscopy can boost the signal. This approach has been investigated using a glioblastoma stem cell EV Raman spectral signature^34^ and to identify MGMT and EGFRvIII from single EVs.^35^

Our multi-omics analysis consistently identified 45 differentially abundant proteins and 20 differentially expressed microRNAs across three independent cohorts. The strong association between the identified proteins and microRNAs with WikiPathways and KEGG pathways, respectively, associated with gliomas and cancer more generally, provides further confidence and reinforces the biological plausibility of our signatures. However, original cellular source of the sEVs remains unclear, with previous research suggesting that a minority of sEVs are directly released from the glioma tumor cells,^36^ with the remainder released from the TME or peripheral sources, including circulating blood cells. Although this does not invalidate the biomarker signature, particularly given the concept of “tumor education” on hematopoietic cells such as platelets^22^ and the glioma microenvironments importance,^37^ it may constrain the extent to which circulating sEV profiling can elucidate intrinsic tumor biology. However, the strong association between our findings and glioma-associated pathways provides reassurance for this approach’s potential. Additionally, the relative contribution of sEV cargo compared to the sEV corona^38^ or non-EV particles as the source of the identified proteins and microRNA in this analysis remains unclear.

Within this study, sEVs’ proteomic profiling revealed downregulation of a network of proteins associated with focal adhesion and integrin binding alongside an increase in proteins associated with the transforming growth factor beta (TGF-β) signaling pathway. Interestingly, previous studies have associated S100A9 with M2-like polarization of tumor-associated macrophages,^39^^,^^40^ identified amyloid accumulation within glioblastoma,^41^^,^^42^ identified THBS1 as a mediator of microtubule formation and tumor invasion,^43^^,^^44^ and associated RELN, a regulator of neuronal migration,^45^ with glioblastoma survival.^46^ Interestingly, we identified downregulation of SVEP1, a relatively understudied protein previously associated with neovascularization in cholangiocarcinoma^47^ and dementia.^48^ Additionally, previous work^49^ has identified upregulation of guanine-nucleotide-binding protein G(i) subunits and ENO1 plus downregulation of C8A across glioblastoma patient and/or mouse model plasma and tumor tissue, echoing the findings in this study. Indeed, of the top 22 proteins, eight and seven proteins were previously identified in nanoparticle-enhanced plasma analysis or tissue analysis, respectively,^49^ providing cross-species validation of these findings and this approach. Further, through unbiased sequencing, we identified several consistently differentially expressed microRNAs with known implications in glioma biology, including downregulation of *miR-139-3p*^50^^,^^51^ and *miR-10a-5p*,^52^ alongside upregulation of *miR-182-5p*.^53^^,^^54^ However, some previously identified proteins and RNA, including the canonical glioblastoma epidermal growth factor receptor variant EGFRvIII protein^55^^,^^56^ and messenger RNA,^57^ were not identified through this analysis, highlighting the need for external controls and multi-center analyses using consistent analytical techniques to facilitate the identification of reproducible differences in a clinical setting.

Machine learning generated highly accurate diagnostic models in the test cohort (AUC:0.957–0.971) with variable external validation performance (accuracy: 40%–100%). This suggests that, in addition to the known challenges with pursuing a machine-learning-based approach when the sample size is limited,^58^ variation between the separate cohorts may have distorted the transferability of some of the identified signatures. For ATR-FTIR spectroscopy, this is in keeping with the reduction in predicted probability of glioma that was identified (Figure 3C). With this analysis, while the analytical processing was identical between the cohorts, we were unable to minimize the impact of any differences in the pre-analytical processing steps when using a limited number of features (Figures 6A and 6B). However, for the proteomics and microRNA analyses, we successfully minimized the impact of sample and technique variation by analyzing only concordant proteins and microRNAs and using z-scores rather than raw abundance values, respectively, as demonstrated by overlapping cohorts on the PCA plots (Figures S5B and S5C).

In this study, we demonstrate 100% accuracy in differentiating glioma patients and controls using a 9-protein (proteomic) and a 10-protein (multimodal) signature in the Longitudinal cohort, albeit with lower accuracy in the Validation cohort. This discrepancy is likely attributable to the different proteomic platforms employed: longitudinal cohort samples were analyzed using the highly reproducible SomaScan Platform (Standard BioTools Inc.), a technique known to have an exceptionally low coefficient of variance (∼5%)^59^ lending itself to inter-study comparisons, whereas Validation cohort samples utilized mass spectrometry, which is more susceptible to inter-batch variation,^60^ underscoring that clinical translation will require standardized pre-analytical protocols and highly reproducible analytical techniques. Further, it is interesting that plasma sEV proteomics have emerged as the most effective and reproducible technique, as evidenced by the perfect differentiation using the proteomic and multimodal models in longitudinal cohort. This finding builds on the recent collective effort to improve and standardize the analysis of plasma proteomics, through analysis of sEVs or nanoparticle enrichment^49^^,^^61^ alongside the emergence of highly reproducible proteomic analysis techniques.^59^^,^^62^ This also raises the possibility that a proteomics-only workflow may retain sufficient discriminatory potential while minimizing the cost requirements of a more comprehensive analysis.

To provide further clinical relevance, we investigated the potential of plasma sEV analysis to differentiate between glioblastoma and IDH^mut^ glioma. While exploratory, clear differences in multimodal sEV analysis were identified, which demonstrates proof of principle for future studies with larger patient cohorts. We also investigated differences in the multi-omic analysis of sEVs from glioma and non-glioma brain tumor patients. In addition to identifying pathways associated with the source of the non-glioma brain tumor samples, the identification of reversed patterns of differential abundance of RELN, THBS1, and SVEP1 within this analysis further highlight that these findings relate to gliomas rather than all brain tumors.

sEV analysis is susceptible to pre-analytical factors including patient co-morbidities, fasting status, and blood collection tubes.^24^^,^^63^^,^^64^ Although guidelines have been proposed,^63^ most biobank samples originate from non-standardized protocols. Therefore, a key strength of our study is the use of independent cohorts with different pre-analytical histories. This approach minimizes confounding pre-analytical influences, as reflected by the segregation of vascular processes among cohort-discordant signals. Furthermore, including a cardiac disease control group mitigated the confounding effects of shared systemic inflammation,^65^ enhancing the specificity of our glioma signature.

In this study, we investigated plasma sEVs, as plasma reduces platelet-derived EVs compared to serum and so is the preferred blood component for sEV biomarker investigations.^66^ However, alternative sources of sEVs have been investigated, such as cerebrospinal fluid, which may be enriched for sEV biomarkers,^67^ and urinary sEVs,^68^^,^^69^ and these approaches may provide complimentary information (Table S15).

### Limitations of the study

This study establishes a multimodal plasma sEV biomarker signature that discriminates glioma patients from controls, albeit certain key questions remain.

While the identified sEV features possess diagnostic utility, making them sufficient as a biomarker, the original cellular source of the sEVs remains unclear. Previous research has suggested that only a minority of sEVs are directly released from the glioma tumor cells,^36^ with the remainder likely to have been released from the TME or peripheral sources such as circulating blood cells. Although this does not invalidate the biomarker signature itself, particularly given the established concept of “tumor education” on hematopoietic cells such as platelets,^22^ it may constrain the extent to which circulating sEV profiling can elucidate intrinsic tumor biology. However, the strong association between the identified proteins and microRNA and glioma-associated pathways provides reassurance for the potential of this approach. Additionally, the relative contribution of sEV cargo compared to the sEV corona^38^ as the source of the identified proteins and microRNA in this analysis remains unclear.

Additionally, due to limited sample availability, statistical analysis was limited to the training cohort, as the validation and longitudinal cohorts were underpowered, and therefore, only an exploratory analysis of the longitudinal assessment capacity of this approach was possible. Further, within this study no correlation of our findings with imaging, pathology including genomics, or other clinical variables, including the influence of sex, was possible. However, our exploratory longitudinal analysis demonstrates the potential of this approach with alterations in a selection of the identified proteins and microRNA alongside the ATR-FTIR spectral signature and sEV characteristics normalizing with treatment and then re-divergence at recurrence. As highlighted above, this in keeping with previous studies have demonstrated that sEV concentration can track tumor volume, with sEV levels decreasing following treatment and increasing with recurrence.^11^^,^^29^^,^^30^

Finally, the statistical power was insufficient for a rigorous comparison of glioma subtypes and for distinguishing gliomas from other brain tumors. Consequently, the analysis of inter-group sEV differences, albeit informative, remains preliminary, and the development of machine learning classifiers for these distinctions was not feasible.

In conclusion, we have developed and externally validated a comprehensive multimodal plasma sEV diagnostic biomarker signature from a 1 mL plasma sample that can differentiate glioma patients from healthy volunteers. While further work to standardize sample processing steps and analysis approaches is required before this work can be translated to clinical practice, this approach could redefine clinical paradigms in neuro-oncology by bridging glioma diagnosis with a minimally invasive liquid biopsy.

## Resource availability

### Lead contact

Further information and requests for resources and reagents should be directed to and will be fulfilled by the lead contact, Georgios Giamas (georgios.giamas@zcmu.edu.cn).

### Materials availability

This study did not generate new unique reagents.

### Data and code availability

• All data are available in the article, from the corresponding author upon reasonable request, from Dxcover Ltd. upon reasonable request, or through the Genomics England^70^ Research Environment.
• Due to data protection requirements, the raw proteomics and microRNA sequencing data cannot be uploaded to a data repository. Summary statistics of all generated data are included within the supplementary tables, and further data can be provided at reasonable request from the corresponding author.
• Additionally, for the training cohort, the raw microRNA sequencing proteomics data have been uploaded to the Genomics England^70^ Research Environment as required for data protection purposes. Research on the de-identified patient data from Genomics England^70^ used can be carried out in the Genomics England^70^ Research Environment subject to a collaborative agreement that adheres to patient-led governance. All interested readers will be able to access the data in the same manner as the authors. For more information, interested readers may contact research-network@genomicsengland.co.uk or access the relevant information on the Genomics England^70^ website: https://www.genomicsengland.co.uk/research.
• All original code, including the feature selection pipeline and the model training parameters, has been deposited at https://github.com/UOSbioinformaticslab/GliomaML/and is publicly available as of the date of publication.
• Any additional information required to reanalyze the data reported in this work paper is available from the lead contact upon request.

## Acknowledgments

The authors thank Nick Hay for his critical discussion and Pascale Schellenberger for her technical support in performing the electron microscopy analysis, and acknowledge the Electron Microscopy Imaging Centre at the 10.13039/501100000838School of Life Sciences, University of Sussex. We thank Lily Hoa for her support with accessing these samples. This research was made possible through access to the samples generated by the 100,000 Genomes Project and access to data in the National Genomic Research Library, which are managed by Genomics England Limited (a wholly owned company of the 10.13039/501100000276Department of Health and Social Care). The National Genomic Research Library holds data provided by patients and collected by the NHS as part of their care and data collected as part of their participation in research. The National Genomic Research Library is funded by the 10.13039/100018336National Institute for Health Research and NHS England. The 10.13039/100010269Wellcome Trust, the 10.13039/501100000289Cancer Research UK, and the 10.13039/501100000265Medical Research Council have also funded research infrastructure. S.D.R. is funded by a University Hospitals Sussex NHS Foundation Trust Medical Doctoral Fellowship and a grant from the My University Hospitals Sussex charity. J.J. is funded by a 10.13039/100018336National Institute for Health Research (NIHR) Clinical Lectureship. The views expressed are those of the author and not necessarily those of the 10.13039/100006662NIHR or the 10.13039/501100000276Department of Health and Social Care. Additionally, this research was partially funded by 10.13039/501100023375Action Against Cancer, grant number ID6292/G1828 awarded to G.G.

## Author contributions

Conceptualization, S.D.R. and G.G.; data collection/analysis/interpretation, S.D.R. (sEV separation, transcriptomics, and statistics), B.T.H. (bioinformatics), M.R.-B. (sEV separation, proteomics, and transcriptomics), O.I. (proteomics), S.P. (ATR-FTIR spectroscopy), P.S.F. (Raman spectroscopy), J.R. (genomics), A.L. (ATR-FTIR spectroscopy), G.A. (ATR-FTIR spectroscopy), M.S. (sEV separation and transmission electron microscopy), W.J. and V.V. (sEV separation), H.J.B. (ATR-FTIR spectroscopy), M.J.B. (ATR-FTIR spectroscopy), M.H. (proteomics), B.T. (transcriptomics and statistics), F.M.G.P. (bioinformatics and genomics), G.G. (sEV separation), and V.V. and C.F. (data analysis); manuscript writing, S.D.R.; funding acquisition, S.D.R., D.G., G.C., and G.G.; scientific and infrastructural support, D.G., G.C., and G.G.; provision of samples, D.S.N., J.J., A.A., J.S.-R., U.L., A.S., A.T., T.S., G.J., R.K., and K.A.; manuscript editing, all authors. All authors have given approval to the final version of the manuscript.

## Declaration of interests

G.G. is the founder and chief scientific officer of Stingray Bio. S.P., A.G., H.J.B., and M.J.B. are employed by Dxcover Ltd.

## STAR★Methods

### Key resources table

**Table:** 

REAGENT or RESOURCE	SOURCE	IDENTIFIER
**Antibodies**		

Anti-CD63	System Biosciences	EXOAB-KIT-1; RRID:AB_2561274
Anti-syntenin	St John’s Laboratory	STJ98556
Anti-apolipoprotein A1	St John’s Laboratory	STJ96804
Anti-albumin	Abcam	ab207327; RRID:AB_2755031
Anti-rabbit IgG HRP linked	Cell Signaling	#7074; RRID:AB_2099233
Anti-mouse IgG HRP linked	Cell Signaling	#7072; RRID:AB_331144
Anti-CD63	Bio-Rad	MCA4754T; RRID:AB_2076616
Anti-CD9	Cell Signaling	D8O1A; RRID:AB_2798139
Anti ALIX	Bio-Rad	MCA2493; RRID:AB_872031
Anti-calnexin	Thermofisher Scientific	MA5-32332; RRID:AB_2809613
Anti-albumin	Bio-Rad	VMA00071
Anti-rabbit IgG HRP linked	Sigma-Aldrich	A6154; RRID:AB_258284
Anti-mouse IgG HRP linked	Santa Cruz Biotechnology, Inc.	sc-516102; RRID:AB_2687626

**Biological samples**		

Training cohort glioma patient plasma samples	This Paper/Genomics England biobank	N/A
Training cohort control plasma samples	Life Science Productions	N/A
Validation cohort brain tumor patient plasma samples	This Paper/Dunedin Brain Tumor Database biobank	N/A
Validation cohort control plasma samples	This Paper/HeartOtago biobank	N/A
Longitudinal cohort plasma samples	This Paper/Neurogenome study biobank	N/A

**Chemicals, peptides, and recombinant proteins**		

Bradford Assay	Bio-Rad™	5000201
RIPA buffer	Sigma–Aldrich	R0278
Triton X-	Sigma–Aldrich	93443
SuperSignal West Pico PLUS Chemiluminescent Substrate	Thermo Fisher Scientific	34580
Clarity Western ECL substrate	Bio-Rad™	1705061

**Critical commercial assays**		

qEV1/35nm size exclusion chromatography columns	IZON	IC1-35
microBCA™ Protein Assay Kit	Thermo Scientific	23235
Dxcover® Liquid Biopsy Platform	Dxcover® Ltd.	N/A
S-35 Trap™ micro spin columns	ProtiFi	C002-MICRO
Oligo R3 resin beads	Thermo Scientific	1-1339-03
0.2 μM PVDF membrane	Corning	C3504
nanoEase M/Z Peptide CSH C18 Column (130 Å, 1.7 μm, 75 μm × 250 mm)	Waters	186008810
SomaScan Platform	Standard BioTools Inc.	N/A
miRNeasy Micro Kit	Qiagen	217084
QIAseq miRNA Library Kit	Qiagen	331505
QIAseq miRNA 96 Index Kit 1L UDI-A	Qiagen	331905
QIAseq miRNA 96 Index Kit 1L UDI-B	Qiagen	331915
P2 XLeap kit	Illumina	20100987
DNBSeq™ profiling	BGI Tech Solutions Ltd.	N/A
nCounter Human V3 miRNA expression panel	Nanostring Technologies	N/A

**Deposited data**		

Training cohort raw proteomics data	This paper	Genomics England Research Environment: https://www.genomicsengland.co.uk/research
Training cohort raw microRNA sequencing data	This paper	Genomics England Research Environment: https://www.genomicsengland.co.uk/research
Validation cohort raw proteomics data	This paper	At reasonable request from the lead contact
Validation cohort raw microRNA sequencing data	This paper	At reasonable request from the lead contact
Longitudinal cohort raw proteomics data	This paper	At reasonable request from the lead contact
Longitudinal cohort raw microRNA sequencing data	This paper	At reasonable request from the lead contact
Machine learning code, including the feature selection pipeline and the model training parameters	This paper	https://github.com/UOSbioinformaticslab/GliomaML/

**Software and algorithms**		

Nanosight NTA 3.2 software	Malvern	N/A
WiRE software	Renshaw	N/A
Progenesis QI for Proteomics v3.0	Nonlinear Dynamics, Waters	N/A
Excel	Microsoft	N/A
R v.4.2.2	Posit Software	N/A
R studio v.2023.06.0	Posit Software	N/A
DIA-NN software	Aptila	https://aptila.bio/
STRING v12.0	STRING	STRING: functional protein association networks
sRNAbench	sRNAtoolbox	sRNAtoolbox
DIANA-miRPath v4.0	DIANA-miRPath	miRPathv4
Prism v.10.4.1	GraphPad	N/A
Clustvis PCA webtool	Clustvis	https://biit.cs.ut.ee/clustvis/

**Other**		

Automated fraction collector (AFC) V1	IZON	AFC-V1
Vivaspin 6 100kDa ultrafiltration filter	Sartorius	VS0641
Nanosight NS300	Malvern Technologies	NS300
Zetasizer Nano ZA	Malvern Technologies	Nano-ZA
Formvar/carbon film coated 200 mesh copper electron microscopy grids	Agar Scientific	S162H
JEOL JEM1400-Plus (120 kV, LaB6) microscope with a Gatan OneView 4K camera	JEOL	N/A
JEOL 2200FS microscope with a C100 imager	JEOL	N/A
GloMax Plate Reader	Promega	GM3000
SpectraMax Microplate Reader	Molecular devices	N/A
iBlot 2 System	Invitrogen	15217995
UVP Chemstudio instrument	Analytik Jena	N/A
Mini Blot Module	Invitrogen	B1000A
ChemiDoc™ gel imaging system	Bio-Rad™	N/A
inVia Qontor Raman microscope	Renishaw	N/A
UltiMate 3000 Rapid Separation liquid chromatography system	RSLC, Dionex Corporation	N/A
Q Exactive Hybrid Quadrupole-Orbitrap	Thermo Fisher Scientific	N/A
*Neo* Vanquish nano-flow liquid chromatography system	Thermo Fisher Scientific	N/A
Orbitrap Exploris 240	Thermo Fisher Scientific	N/A
NextSeq1000	Illumina	N/A
nCounter GEN2 digital analyser	Nanostring Technologies	N/A

### Experimental model and study participant details

#### Patients and clinical samples

A total of 206 plasma samples from 159 individuals within three independent cohorts (training, validation, and longitudinal cohorts) were identified from three separate biobanks (Table S1). Samples were identified based on their availability at each of the three biobanks. All samples were provided to their respective biobank with written informed consent.

For the training cohort, *n* = 56 glioma patients, *n* = 48 controls), pre-operative plasma from glioma patients recruited to the 100K Genome Project with available plasma samples were provided from the Genomics England^70^ biobank (REC:20/EE/0035). Ethical approval was provided by the NHS Health Research Authority (REC:18/EM/0071) and the Genomics England^70^ Research Committee (RR295). Demographics and whole genome sequencing results were available through the Genomics England^70^ Research Environment. Patients were re-classified according to the WHO CNS 5 classification^2^ based on the presence of an IDH1/IDH2 mutation identified within their whole genome sequencing. Gender- and age-matched (≤±5 years, Table S2) healthy volunteers’ plasma were purchased from a commercial biobank (Life Science Productions).

For the validation cohort, (*n* = 13 glioma patient, *n* = 16 non-glioma patients, *n* = 20 controls), pre-operative plasma samples from available brain tumor patients (glioma, meningioma, brain metastases, lymphoma, Table S1) were provided by the Dunedin Brain Tumor database, while cardiovascular disease control samples from the HeartOtago database. Ethical approval was provided by the Northern B Health and Disability Ethics Committee (Ethics ref. 2024 a.m. 19014).

For the longitudinal cohort (*n* = 6 glioblastoma patients), plasma samples at defined time points from available IDH^wt^ glioblastoma patients’ undergoing standard of care conventionally fractionated chemoradiotherapy (60 Gray in 30 fractions with concurrent and adjuvant temozolomide) were provided by the Neurogenome study^15^ biobank (H-21023801). Ethical approval was provided by the National Danish Ethics Committee (H-21023801).

### Method details

#### Blood sample processing

The blood collection and processing reporting has been guided by MIBlood-EV recommendations.^71^ For the training cohort, blood was collected from fasting donors in K_2_EDTA (*n* = 49 glioma and all healthy volunteers), Streck (*n* = 5 glioma), or Lithium Heparin (*n* = 2 glioma) blood tubes, spun down to plasma (1300-2000x *g* for 10 min) within 6 h (≤72 h for Streck), and stored as 1 mL aliquots at −80°C. For the validation cohort, blood was collected from donors after 6 h of fasting in EDTA blood tubes, spun down to plasma (2000x *g* for 10 min) within 1 h, and stored as 1 mL aliquots at −80°C. While for the longitudinal cohort, blood was collected from non-fasting donors in two 10 mL Streck tubes, spun down to plasma (2250x *g* for 10 min then 16,000x *g* for 10 min at 20°C) within 24–72 h, and stored as 1 mL aliquots at −80°C.

#### Separation and concentration of sEVs

Plasma samples from the training and longitudinal cohorts were processed to separate sEVs at the University of Sussex, United Kingdom (Sussex), while the validation cohort samples were processed to separate sEVs at the University of Otago, New Zealand (Otago).

sEVs were separated from 1 mL plasma at Sussex as previously described.^16^ Briefly, visibly non-haemolysed plasma samples were defrosted on ice and then underwent centrifugation at 1500× *g* for 10 min at room temperature and the supernatant was centrifuged at 10,000× *g* for 10 min at room temperature to remove cellular debris. The cleared supernatant was collected and topped up to 1000 μL with sterile-filtered PBS. At Otago, the same sEV separation protocol was followed with the addition of a 0.22 μm syringe filtration step before size exclusion as previously described.^72^

Size exclusion chromatography columns (Izon qEV1/35 nm) were placed in an automated fraction collector (Izon), following which they were drained and then flushed with sterile filtered (0.2 μm) PBS. The sample was loaded into the size exclusion chromatography column followed by layering 8 mL sterile-filtered PBS. The buffer volume was defined as 4.7 mL while the fraction size was defined as 700 μL. As per the manufacturer’s recommendations, and previous characterisation experiments,^16^ the buffer volume was allowed to pass through the column and then the required fractions collected and pooled (Sussex: fraction 1–4, Otago: fraction 2–3) to obtain a sample balancing EV recovery with sample purity.

The combined sample (2.8 or 1.4 mL) was concentrated to 100 μL using a 100 kDa molecular weight ultrafiltration filter (Vivaspin 6, Sartorius) and centrifugation at 4000× *g* (Mega Star 1.6R, VWR). sEV samples were stored at −80°C.

#### Characterisation of sEVs

Following the minimal information for studies of extracellular vesicles guidelines,^73^ the sEVs were characterised by several orthogonal methods to confirm their separation as previously described.^16^

sEV concentration and size were determined by nanoparticle tracking analysis using a NanoSight NS300 equipped with a 488 nm blue laser (Malvern Technologies) in Sussex. The sEV samples were diluted 1:1000 in sterile-filtered PBS to be within the detectable range of the NS300. Five measurements of 60 seconds each were recorded using a camera level set close to maximum (14). Analysis was performed automatically using the Nanosight NTA 3.2 software (Malvern Instruments) using a consistent detection threshold of 5. The following conditions were applied for the analysis at the Nanosight instrument: temperature was 20–25°C; viscosity was ∼0.98 cP; camera type was sCMOS; and the syringe pump speed was set to 70 AU. At Otago, particle size and concentration were determined by dynamic light scattering using a Zetasizer Nano ZA (Malvern Technologies) as previously described.^72^ The sEV samples were diluted 1:20 in PBS to prevent sample clogging, while a concentration gradient was created using 100 nm calibration particles (Izon).

Particle morphology was visualised by transmission electron microscopy. At Sussex, samples were diluted in sterile-filtered PBS to a particle concentration of ∼5×10^10^/mL. Subsequently, 4 μL was applied to a formvar/carbon film coated 200 mesh copper EM grid (Agar Scientific cat no. S162H) that had been previously treated with a glow discharger (PELCO easiGlow) for 1 min. The samples were incubated for 30 s then washed with PBS and stained with filtered 1% Uranyl Acetate for 30 s and allowed to dry. The grids were visualised using a JEOL JEM1400-Plus (120 kV, LaB6) microscope (JEOL Ltd., Welwyn Garden City, UK) equipped with a Gatan OneView 4K camera at ×10,000 and ×30,000 magnification. In total, 10–15 representative images per grid were taken. At Otago, samples were diluted in sterile-filtered PBS to a particle concentration of ∼2.5×10^10^/mL. Subsequently 5 μL of sample were applied to a carbon coated 200 mesh copper EM grids that had been previously treated with glow discharge for 30 s. The samples were incubated for 1 min then blotted dry via capillary action using filter paper. Samples were then stained with 10 μL of pH 7, 1% phosphotungstic acid (PTA), which was immediately blotted away. Once dried grids were visualised using a JEOL 2200FS microscope. The grids were imaged using a C100 imager magnifications ranging from ×10,000 to ×100,000 magnification.

Additionally, immunogold labeling of CD81 was performed at Otago. The samples were fixed 1:1 in 2% paraformaldehyde for 5 min before application to grids. Samples were incubated on grids for 10 min, washed with PBS, and then blocked in 1% BSA for 30 min. Grids were treated with 50 μL of antibody diluted 1:100 in PBS containing 0.1% BSA overnight at 4^o^C and then washed in 50 μL of PBS containing 0.1% BSA. Grids were transferred to secondary antibodies (anti-mouse IgG conjugated to 9–11 nm gold particles) diluted 1:100 in 0.1% BSA PBS for 1 h and then washed in 50 μL of PBS. Negative staining was then carried out using PTA as previously described.

Protein quantification was performed using the microBCA Protein Assay Kit (Thermo scientific) at Sussex or Quick Start Bradford protein assays (Bio-Rad, USA) at Otago. Briefly, at Sussex 2 μL of sEVs were lysed in 5 × RIPA buffer for 10 min in technical duplicate in a 96-well microplate. Subsequently, 100 μL of BCA reagent was added to each sample. Additionally, a standard BSA curve (serial dilutions from 10 μg to 0.078125 μg) and a RIPA control sample were incubated with 100 μL of BCA reagent. The plate was incubated at 37°C in the dark for 2 h. Absorbance was measured at 560 nm (GloMax, Promega). Protein concentration was determined from the BSA standard curve and incorporated the influence of the RIPA buffer on the measured absorbance. At Otago, 2 μL of concentrated sEVs were lysed in in a final Triton X- concentration of 0.01% (partial lysis) for 20 min in technical triplicates in a 9-well microplate. Subsequently, 100 μL of Bradford reagent was added to each sample. Additionally, a standard BSA curve (serial dilutions from 20 μg to 0.5 μg) and a Triton X- control sample were incubated with 100 μL of Bradford reagent. The plate was incubated at 37°C for 5 min. Absorbance was measured at 595 nm (SpectraMax, Molecular devices). Protein concentration was determined from the BSA standard curve and incorporated the influence of the Triton X- on the measured absorbance.

Protein expression of known EV surface markers (CD63 and/or CD9), EV cargo markers (syntenin or ALIX) and commonly co-separated proteins (albumin and apolipoprotein A1 (ApoA1) or calnexin) were investigated by Western blot to confirm enrichment of sEVs in the sample. At Sussex, sEV samples (20 μg as quantified by microBCA) and unprocessed plasma (20 μg or 4 μg as quantified by microBCA) were lysed in 5 × Laemmli sample buffer (containing 1.5 M Tris–HCl, Glycerol, 20% sodium dodecyl sulfate (SDS), 0.25% bromophenol blue and 1 M dithiothreitol), boiled at 95°C for 10 min, and resolved on SDS/PAGE gradient gels (4–12%). Transfer was performed using the iBlot 2 system (Invitrogen). Primary antibodies: anti-CD63 (1:1000, System Biosciences EXOAB-KIT-1), anti-syntenin (1:1000, St John’s Laboratory STJ98556), anti-apolipoprotein A1 (1:1000, St John’s Laboratory STJ96804), anti-albumin (1:2000, Abcam ab207327). Secondary antibodies: Anti-rabbit IgG HRP linked (1:5000, Cell Signaling #7074) and Anti-mouse IgG HRP linked (1:5000, Cell Signaling #7072). Membranes were incubated with SuperSignal West Pico PLUS Chemiluminescent Substrate (Thermo Fisher Scientific) and imaged with a UVP Chemstudio instrument (Analytik Jena). At Otago, 1 μg sEV samples and unprocessed plasma (as quantified by Bradford assay) were lysed in 6x loading buffer (containing 1.5M Tris HCL ph8.8, Glycerol, 20% SDS, 0.5% betamercaptoethanol, and 0.05% bromphenol blue), boiled at 95°C for 10 min, and resolved on SDS/PAGE gradient gels (4–12%). Transfer was preformed using the Mini Blot Module (Invitrogen). Primary antibodies: anti-CD63 (1:500, Bio-Rad MCA4754T), anti-CD9 (1:500 Cell signaling D8O1A), anti-ALIX (1:1000 Bio-Rad MCA2493), anti-Calnexin (1:1000 Thermofisher Scientific MA5-32332), Anti-albumin (1:1000 Bio-Rad VMA00071). Secondary antibody: Anti-Rabbit IgG HRP linked (1:5000, sigma-Aldrich A6154) and Anti-mouse IgG HRP linked (1:5000 Santa Cruz Biotechnology, Inc. sc-516102). Membranes were incubated in Clarity Western ECL substrate (Bio-Rad 1705061) and imaged with a ChemiDoc gel imaging system (Bio-Rad).

#### EV-TRACK Registration

We have submitted all relevant data of our experiments to the EV-TRACK knowledgebase^74^ (EV-TRACK ID: EV250034).

#### ATR-FTIR spectroscopy and Initial data processing

sEV samples from the training and longitudinal cohorts were analyzed using the Dxcover Liquid Biopsy Platform (Dxcover Ltd.) as previously reported.^14^ The sEV samples were allowed to thaw for up to 30 min at room temperature (18°C–25°C) and inverted three times to ensure sufficient mixture and thawing. Each sample was prepared by pipetting 6 μL of sEVs onto three sample wells of the Dxcover Sample Slides (Dxcover, Glasgow, UK). Prepared slides were placed in a drying unit incubator (Thermo Fisher Heratherm, GE) at 35°C for 10 min, to control the dehydration process of the sEVs. The dried sample slides were loaded on to the Dxcover Autosampler (Dxcover, Glasgow, UK) and prepared for spectral collection. In this study, a PerkinElmer Spectrum 2 FTIR spectrometer (PerkinElmer, USA) was used to generate spectral data (16 co-added scans at 4 cm^−1^ resolution, with 1 cm^−1^ data spacing). A total of three spectra were collected for each sample well, resulting in nine replicates per patient and a background measurement was obtained from the blank well of the Sample Slide.

A sequence of pre-processing steps were applied to the raw spectra. First, the spectra were truncated to the wavenumber range of 3500 cm^−1^-1000 cm^−1^. They were then aligned to an appropriate reference using Extended Multiplicative Signal Correction (EMSC). Finally, the silent wavenumber region (2799 cm^−1^-1801 cm^−1^, inclusive) was removed, as no biologically relevant molecular bonds are excited in this frequency range. Molecular assignment was performed based on previously published values^17^ (Table S3).

#### Raman Spectroscopy and Initial data processing

For a subset of the training cohort, 10 μL of each sEV sample was pipetted onto a mirrored stainless-steel slide and dried at 37^o^C (5 min). Raman spectral acquisitions were taken manually with a confocal Raman microscope inVia Qontor (Renishaw) with calibration using the 520 cm^−1^ single peak of silicon (1.0 s exposure time). All spectra were recorded for the fingerprint region (wavenumber range 616 cm^−1^-1719 cm^−1^) with a 50x objective and a 785 nm laser. Spectra measurements were taken at an integration exposure time of 10 s (6 accumulations).

WiRE software (Renishaw) was used for automatic cosmic ray removal and baseline subtraction. Each dataset was processed in a matrix format of wavenumber points with their corresponding intensity for each acquisition. Intensity values of average merged spectra (12 areas per sample) were normalised to between 0 and 1 using a min-max formula. Molecular peak assignment was performed based on previously published values^18^ (Table S3).

#### Proteomics and Initial data processing

For the training cohort, suspension trapping (S-trap) and trypsin digestion was performed on purified sEV samples as previously described.^75^ A volume of sEVs equivalent to a total of 10 μg of protein (as quantified by microBCA) was mixed with 10 μL of lysis buffer (50 mM triethylammonium bicarbonate (TEAB) pH 7.5, 5% SDS) and incubated at 4°C for 1 h sEV samples were reduced with 5 mM DTT at 60°C for 10 min while being centrifuged at 800 rpm and then alkylated with 15 mM iodoacetamide (IAM) in the dark for 30 min. DTT was added as before to quench the IAM and the samples cleared by centrifugation at 14,000x *g* for 10 min at 4°C. The supernatant was transferred to a fresh tube and acidified with 1.2% w/v phosphoric acid. S-trap binding buffer (90% methanol, 100 mM TEAB, pH 7.1) was added as such to increase the sample volume by 6-fold and the samples loaded onto S-35 Trap micro spin columns (ProtiFi). Columns were centrifuged at 4,000x *g* for 2 min and the flow through discarded. Columns were washed once with methyl *tert*-butyl ether in methanol (10:3) and then a further four times with S-trap binding buffer and the flow through discarded after each wash. In column digestion was performed by incubation with trypsin (0.05 μg/μL) for 1 h at 47°C and the resultant peptides eluted by addition of S-trap digestion buffer (50 mM TEAB pH 8.5). Peptides were further eluted with 0.1% formic acid (FA) followed by 30% acetonitrile in 0.1% FA and subsequently desalted using Oligo R3 resin beads (ThermoScientific) in a 96-well S-trap filtration plate with a 0.2 μM PVDF membrane (C3504, Corning). Beads were washed twice in 0.1% FA in water and the plate centrifuged at 200x *g* for 1 min. Samples were loaded onto the plate and incubated at room temperature for 5 min while being centrifuged at 500 rpm. The plate was centrifuged as before and the waste discarded. Beads were washed twice and the peptides finally eluted with 0.1% FA in 30% acetonitrile and lyophilised using a MiVac Centrifugal Quattro Concentrator (Genevac).

Dried peptides were resuspended in 10 μL of 0.1% FA in 5% acetonitrile and analyzed by label-free LC-MS/MS using an UltiMate 3000 Rapid Separation LC (RSLC, Dionex Corporation) coupled to a Q Exactive Hybrid Quadrupole-Orbitrap (Thermo Fisher Scientific) mass spectrometer. Peptide mixtures were separated using a gradient from 95% A (0.1% FA in water) and 5% B (0.1% FA in acetonitrile) to 18% B, in 34.5 min, 27% at 42.5 min, and 60% at 43.5 min, at a flow rate of 300 nL/min, using a 75 mm × 250 μm inner diameter 1.7 μM CSH C18 analytical column (Waters). Peptides were selected for fragmentation automatically by data dependant analysis (DDA). Data was acquired for 60 min in positive mode.

Raw data was processed using Progenesis QI for Proteomics software (Nonlinear Dynamics, Waters). The produced XML file was imported into Progenesis QI v3.0 where the program automatically selected a reference run in which all other runs were aligned to. MS/MS peaks were searched against the human (*Homo sapiens*) proteome (Uniprot database) with a local Mascot server (Matrix Science) using the following search parameters: fixed modification: carbamidomethyl (C), variable modification: oxidation of methionine (M), trypsin as the digestion enzyme with maximum missed cleavages set to one, +2 and +3 peptide charges, 15 mmu precursor mass tolerance, 8 ppm fragment mass tolerance and ESI-QUAD-TOF selected as the instrument. The software mapped peptides to features with a peptide-spectrum match score >20, applying a 1% false discovery rate (FDR) filter to all significant (*p* < 0.05) peptide-spectrum match matches. The resulting protein lists with one-way ANOVA *p*-value, q-value, maximum fold change, and normalised protein abundance were exported from Progenesis and further analyzed using Excel (Microsoft), R (v. 4.2.2), and RStudio (v. 2023.06.0, Posit Software).

For the validation cohort, protein extraction and digestion were also performed using the micro S-Trap (Protifi) procedure essentially as per the manufactures protocol. Briefly, proteins from a volume of sEVs equivalent to a total of 4 μg were solubilised in 5% SDS in 100 mM TEAB, pH 7.9 and then reduced and alkylated with 5 mM tris(2-carboxyethyl)phosphine (TCEP) and 10 mM IAM respectively. Extracted proteins were purified on micro S-Trap units and digested with trypsin (sequencing grade, Promega) at an enzyme to protein ratio of 1:25. Digested protein samples were further purified by solid phase extraction on peptide desalting columns (Thermo Scientific) following the manufacture’s protocol. Small aliquots of representative sample were taken and pooled into a quality control sample.

Samples were analyzed by data-independent acquisition (DIA) mass spectrometry on an Orbitrap Exploris 240 (Thermo Scientific) coupled to a *Neo* Vanquish nano-flow liquid chromatography system. For each run 4 μg of digested protein were loaded on an emitter tip column (75 μm × 20 cm) packed inhouse with Luna C-18 material (100 Å pore size, 3 μm bead size) and separated by a gradient from mobile phase A (0.05% FA in water) to mobile phase B (98% acetonitrile, 0.05% FA in water) over a total run time of 120 min at a flow rate of 400 nL/min. The mass spectrometer was operated in DIA mode to acquired one full MS scan in the mass range of 400–1500 m/z at a resolution of 60,000 followed by 24 DIA-fragment ion scans covering the mass range of 400–1500 m/z. The fragment ion spectra were acquired at a resolution of 15,000 with an accumulation time of 75 ms and a normalised automated gain control of 1000, resulting in a total cycle time of 2 s. Raw data were analyzed using the DIA-NN software (https://aptila.bio/) and then exported and analyzed using Excel (Microsoft).

While for the longitudinal cohort, the samples were analyzed using the aptamer-based proteomics SomaScan Platform (Standard BioTools Inc.), a highly multiplexed aptamer-based proteomic technology capable of making over 11,000 simultaneous protein measurements, according to the manufacturer’s recommendations as previously described.^76^ Additionally, sEV samples from gender- and age-matched (≤±5 years) healthy controls within the training cohort (*n* = 6) were analyzed in addition to allow the comparison of protein abundance changes within the longitudinal cohort.

Briefly, sEV samples were lysed using the recommended lysis buffer (120mm NaCl, 5mM KCl, 5mM MgCl_2_, 40mM HEPES pH 7.5, 0.05% Tween 20, 1% NP40 (v/v), 0.5% sodium deoxycholate (w/v)) and diluted to a concentration of 200 μg/mL. The lysed samples were subsequently shipped on dry ice to Somalogic (Boulder, Colorada, USA) and then run on the SomaScan Platform using a custom 100 marker panel (Table S4) on the 11K platform as previously described.^77^

Briefly, sEV samples are incubated with chemically modified DNA aptamers that form complex three-dimensional shapes which bind to epitopes on their target protein. After several washing and enrichment steps, the amount of the available protein epitope is readout by hybridizing the aptamer reagents in the assay eluate to complementary sequences on a DNA microarray and the fluorescence measured as relative fluorescence units (RFU).

Hybridisation normalisation, plate calibration and scaling alongside intrastudy median signal normalisation, and quality control checking was performed by Somalogic on all samples as previously described.^77^^,^^78^ Briefly, hybridisation normalisation was performed through the addition of control reagents prior to imaging to mitigate variation arising from the readout steps, within-plate and across-plate technical variation was controlled for by the inclusion of five pooled reference standards and three buffer only control samples on each plate following which the samples were further normalised using median signal normalisation to provide the output RFU values, while quality control checking assessed the values used for normalisation against standardised SomaLogic quality control criteria.

To interrogate the functionality of the identified proteins, STRING v12.0 (String: Functional Protein Association Networks)^79^ was used to perform protein association networks and pathway analysis using only those associations identified from curated databases or with experimental evidence.

#### microRNA analysis and Initial data processing

For the training and longitudinal cohorts, microRNA was extracted from 40 μL of each sEV sample using the miRNeasy Micro Kit (Qiagen) according to the manufacturer’s protocol. Briefly, the sEV sample was incubated with 1000 μL of ice-cold QIAzol Lysis Reagent for 5 min at room temperature. Subsequently, add 200 μL of chloroform, mix vigorously for 15 s and incubate for 3 min. Centrifuge at 12,000x g for 15 min at 4°C. Transfer the aqueous phase to a new tube and add 1.5 volumes of 100% ethanol and mix thoroughly. Add 700 μL of sample into the provided spin column and centrifuge at 15,000x *g* for 15 s. Re-add the flow through to the spin column, repeat the centrifugation, and then discard the flow-through. Repeat until all the sample has been processed through the spin column twice. Wash the spin column using the buffers provided and a 15 s centrifugation at 12,000x *g* (700 μL buffer RWT then 500 μL buffer RPE) and then wash with 500 μL ice-cold 80% ethanol with a 2 min centrifugation at 12,000x g. Dry the spin column by centrifugation at 12,000x *g* for 5 min, add 14 μL RNAse free water, and then elute the sEV RNA by centrifugation at 12,000x *g* for 1 min.

For the training cohort, the QIAseq miRNA Library Kit (Qiagen) was used with 5 μL of sEV RNA and the QIAseq miRNA 96 Index Kit 1L UDI-A/B (Qiagen) to prepare microRNA libraries for sequencing with minor modifications to the manufacturer’s protocol as previously reported, but with 21 amplification cycles.^80^ The following modifications to the manufacturer’s protocol were used: (1) the 3′ adapter and the primer for reverse transcription were used at a 1:20 dilution, (2) the 5′ adapter was used at a 1:10 dilution, (3) all bead-washing steps were done using 500 μL of 80% ethanol rather than the recommended 200 μL, and (4) at the library amplification step, denaturation, annealing and extension were performed for 21 polymerase chain reaction (PCR) amplification cycles. Quality control was performed by Tapestation 4150 (Agilent) for accurate library sizing and Qubit dsDNA Assay Kit (Thermo Fisher Scientific) for library quantification. The libraries were then pooled at equimolar concentrations for sequencing, spread across two flow cells with equivalent numbers of glioma patients’ and healthy volunteers’ samples per run. The pooled libraries were subject to 75 base pair (bp) single-end sequencing using the P2 XLeap kit on a NextSeq1000 (Illumina).

For the longitudinal cohort, plus the matched healthy control sEV samples (*n* = 6), following microRNA extraction, small RNA sequencing was performed by BGI Tech Solutions Ltd. using their DNA Nanoball sequencing technology (DNBSeq) as previously described.^81^ Briefly, 6.5 μL of sEV RNA underwent 3′ end ligation, followed by unique molecular identifier (UMI) addition, and then 5′ end ligation. Synthesis of cDNA was performed using UMI labeled primers followed by PCR amplification. Following library fragment selection (110–140 bp) and quality control, the cDNA was circularised and then sequenced using DNBSeq. Filtering using SOAPnuke^82^ was performed on raw data with adapter sequences or low-quality sequences, followed by trimming of adapters, removal of low quality reads, and filtering of read length (keep reads with 15–44 bp), resulting in the clean data output.

For the validation cohort (*n* = 6), microRNA analysis was performed using an nCounter microRNA array (Nanostring) as previously described.^83^ Briefly, microRNA was extracted from 700 μL of sEV samples using the miRNeasy mini kit (Qiagen) as per the manufacturer’s instructions and quality control was performed using a NanoDrop-1000 spectrophotometer (NanoDrop Technologies) and an Agilent Bioanalyzer. Subsequently, 100 ng of sEV miRNA was added to the miRNA-tag ligation reaction, and then 10 ng of miRNA was hybridised with 827 miRNA-specific DNA probes that are part of the nCounter Human V3 miRNA expression panel and imaged using the GEN2 digital analyser (NanoString Technologies) to quantify the number of microRNAs in each sample. A set of six positive control probes, 8 negative control probes, 3 positive ligation probes, and 3 negative ligation probes were included. miRNA values were normalised according to the mean positive control value.

For the training and longitudinal cohorts, alignment to known human microRNA was performed via sRNAbench (sRNAtoolbox)^84^ using the miRbase release 22.1 reference database,^85^ while differential expression was performed using sRNAde and unique reads only (sRNAtoolbox),^84^ with microRNAs determined as differentially expressed between conditions when adjusted *p* < 0.05. Additionally, for all the cohorts, target pathways of the differentially expressed microRNA were identified using DIANA-miRPath v4.0 (miRPathv4)^86^ using only those targets from the miRTarBase 2022 database with strong evidential evidence.

#### Machine-learning algorithms including feature selection

Given the complexity of the ATR-FTIR spectral data, a model of glioma prediction was generated encompassing the whole spectrum. Initial model generation was performed using partial least-squares regression and distance weighted discrimination models. Models were trained following a nested cross-validation approach (outer cross-validation: randomly stratified 70:30 split for training:testing, repeated 51 times; inner cross-validation: 5-fold cross-validation in the training set). From the ensemble of 51 models, consensus was determined through majority voting across the 9 spectra and then across the ensemble. The final ensemble receiver operating characteristic (ROC) curve was generated by further consensus voting across the models. Spectra measured from the same sample were allowed to appear in a single subset for both outer and inner cross-validation, to avoid information leakage. The final model was used to generate prediction probabilities for a glioma/IDH^wt^ glioblastoma diagnosis.

Additionally, three independent machine learning models were trialled to develop the most accurate classification system. Random Forest Models (RF), k nearest neighbor (kNN), and Extreme Gradient Boosting (XGBoost) algorithms, as described in the sci-kit learn package,^87^ were employed in Python to develop signatures, including the individual -omics/spectral data and the integrated multimodal data, that differentiated glioma patients from healthy volunteers.

To minimise the influence of batch effects between different cohorts, technical variation related to sample collection and processing differences rather than from true biological differences, z-scores were calculated for each of the variables within the three cohorts. Principal Component Analysis (PCA) was used to confirm the effectiveness of using the *Z* score to minimise the impact of a batch effect in the combined cohort datasets by visualising the clustering of samples. Further, to address the challenge of high dimensionality given the limited sample size, feature selection was applied to retain the most relevant features for classification. The recursive feature elimination (RFE) method from scikit-learn^87^ was utilized to select the top 10 most important features per dataset, aiming to enhance model performance by reducing noise while preserving discriminative information.

Model training was conducted using a 5-fold cross validation approach to ensure robustness, while hyperparameter tuning was performed using grid search to optimise model performance. The dataset was randomly stratified (80:20) into training and validation sets to maintain class balance. Model performance was evaluated based on the mean area under the ROC curve (AUC) and standard deviation (SD) across the 5-fold cross validation. Additionally, mean accuracy, mean precision, mean recall, F1-score, and their respective SD were calculated. The model yielding the highest mean AUC with the lowest standard deviation was selected as the best performing model for each dataset.

To assess whether the model was overfitting or capturing biologically meaningful patterns, a class shuffling analysis was conducted by randomly assigning 50% of each class’s labels. Subsequently the best performing model was retrained on the shuffled dataset to assess whether the model had learned true discriminative features, in which case performance would be expected to degrade significantly. Following class shuffling, an AUC of approximately 0.5 indicates that the model can no longer distinguish between classes and was performing at a chance level.

### Quantification and statistical analysis

Results are reported as the mean ± standard error of the mean (SEM). Welch’s unpaired *t* test or one-way ANOVA with post-hoc Tukey were used to assess differences in sEV features, while ROC curve analysis assessed model performance using GraphPad Prism (v.10.4.1). Differences were determined at *p* < 0.05. PCA was performed using ClustVis webtool (https://biit.cs.ut.ee/clustvis/). Different significance levels were reported as follows: not significant (ns) *p* ≥ 0.05, ∗*p* < 0.05; ∗∗*p* < 0.01; ∗∗∗*p* < 0.001; ∗∗∗∗*p* < 0.0001.

## Supplemental information

Document S1. Figures S1–S6

Table S1. Summary of samples and analysis techniques performed, related to STAR methods

Table S2. Summary of glioma patients’ and healthy volunteers’ demographics in the training cohort, related to STAR methods

Table S3. Table of molecular assignments for the 10 most important ATR-FTIR spectral peaks and the molecular assignments for the 10 most important Raman spectral peaks identified through recursive feature elimination, related to STAR methods

Table S4. Summary of the targets of the SOMAmers used within the custom 100-marker panel, related to STAR methods

Table S5. Summary of the identified differentially abundant proteins between glioma patients’ plasma sEVs and healthy volunteers’ plasma sEVs in the training cohort (adjusted *p* < 0.05), related to Figure 4

Table S6. Summary of the 502 proteins present in at least 67% of samples within the validation cohort following removal or common contaminant proteins (keratins, immunoglobulin variants, apolipoproteins, and albumin), related to Figure 4

Table S7. Table of the proteins identified within the samples of both the training and validation cohorts to demonstrate the concordance in differential abundance between cohorts, related to Figure 4

Table S8. Table comparing the differential protein abundance pattern between healthy controls and the pre-operative glioblastoma patient samples in the longitudinal cohort with the differential protein abundance patterns identified within the training and validation cohorts, related to Figures 4 and 7

Table S9. Summary of the identified differentially expressed microRNA between glioma patients’ plasma sEVs relative to healthy volunteers’ plasma sEVs in the training cohort (*q* < 0.05), related to Figure 5

Table S10. Table of the microRNA identified within the samples of both the training and validation cohorts to demonstrate the concordance in differential abundance between cohorts, related to Figure 5

Table S11. Summary of the microRNA consistently identified within the longitudinal cohort samples, related to Figure 5

Table S12. Table comparing the differential microRNA expression pattern between the healthy controls and the pre-operative glioblastoma patient samples in the longitudinal cohort with the differential microRNA expression patterns identified within the training and validation cohorts, related to Figures 5 and 7s

Table S13. Table of model performance characteristics in the training cohort for the different generated models, related to Figure 6 The most effective models are highlighted in bold. ATR-FTIR, attenuated total reflection Fourier transform infrared; RF, Random Forest; kNN, K nearest neighbor; XGB, extreme gradient boosted; AUC, area under the curve; SD, standard deviation

Table S14. Table of model performance characteristics in the internal and external validation cohorts for the optimal models alongside the class shuffling analysis, related to Figure 6 ATR-FTIR, attenuated total reflection Fourier transform infrared; RF, Random Forest; kNN, K nearest neighbor; XGB, extreme gradient boosted; AUC, area under the curve; SD, standard deviation

Table S15. Table of overlapping plasma sEV proteins identified in this study and urine sEV proteins identified by Hallal et al.

Document S2. Article plus supplemental information
