# Supplementary material for: Combined multi-omics and multi-spectral profiling of plasma extracellular vesicles reveals liquid biopsy biomarkers for glioma diagnosis
Source: Cell Rep Med. 2026 Apr 17;7(5):102744. doi: 10.1016/j.xcrm.2026.102744 (PMC13198237; doi:10.1016/j.xcrm.2026.102744)
Supplement: Document S1. Figures S1–S6 [file mmc1.pdf]

Cell Reports Medicine, Volume *7*

# Supplemental information

# Combined multi-omics and multi-spectral profiling of plasma extracellular vesicles reveals liquid biopsy biomarkers for glioma diagnosis

**Stephen David Robinson, Biniam Tsegay Haile, Matthew Reily-Bell, Olivia Iwanowytsch, Siobhan Palmer, Dorte Schou Nørøxe, Panagiota S. Filippou, Joanna Renaut, Alan Lazarus, Georgios Antoniou, Mark Samuels, Viviana Vella, Chrysa Filippopoulou, William Jones, Josephine Jung, Xiaoou Li, Nan Ji, Yang Zhang, Aleena Azam, Jane Skjoeth-Rasmussen, Ulrik Lassen, Adriana Saraiva, Ahmad Taha, Tania Slatter, Greg Jones, Rajesh Katare, Holly J. Butler, Matthew J. Baker, Marilena Hadjidemetriou, Duncan Gilbert, Benjamin Towler, Keyoumars Ashkan, Giles Critchley, Frances M.G. Pearl, and Georgios Giamas**

SUPPLEMENTARY FIGURES:

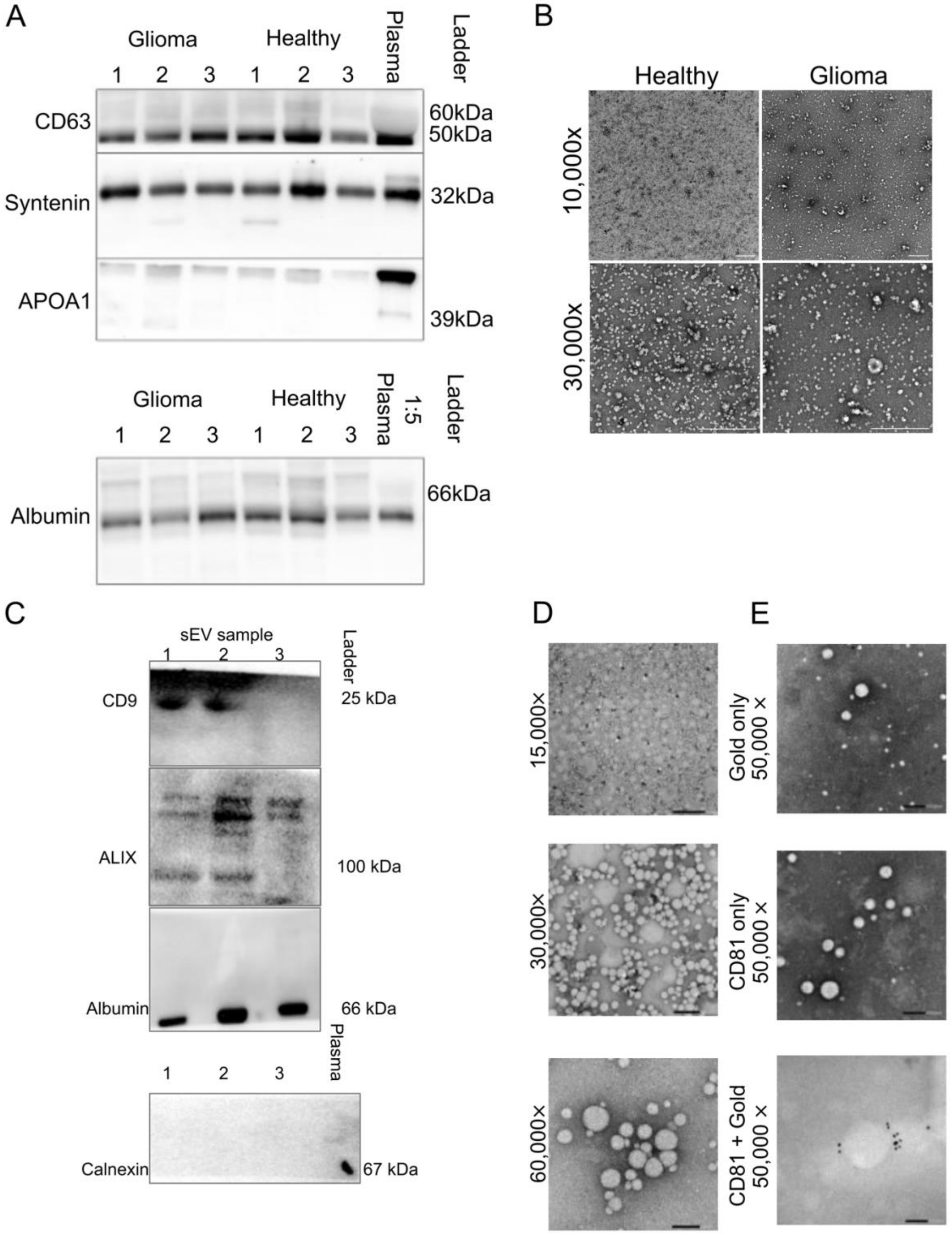

**Supplementary Figure 1. Sample characterisation of plasma sEVs according to the minimal information for studies of extracellular vesicles guidelines[17]. Related to Figure 2 and STAR methods.**

Uncropped Western blot images demonstrating the enrichment of sEV surface markers (CD63, CD9), sEV cargo makers (Syntenin, ALIX), and the depletion of common co-separated proteins (ApoA1, albumin, calnexin) from sEV samples or unprocessed plasma sample at **A**) Sussex (20 µg samples or 4 µg unprocessed plasma for albumin assessment) and **B**) Otago (1 µg samples). Transmission electron microscopy using wide-field (10,000-15,000x magnification) and near-field (30,000-60,000x magnification) images demonstrate the expected morphology of sEVs at **C**) Sussex and **D**) Otago. **E**) Immunogold labelling demonstrates the association between the identified particles a known sEV surface marker (CD81). White scale bars 500 nm, Black scale bars 320 nm (15,000x magnification), 160 nm (30,000 magnification), 100 nm (50,000x magnification) or 80 nm (60,000 magnification).

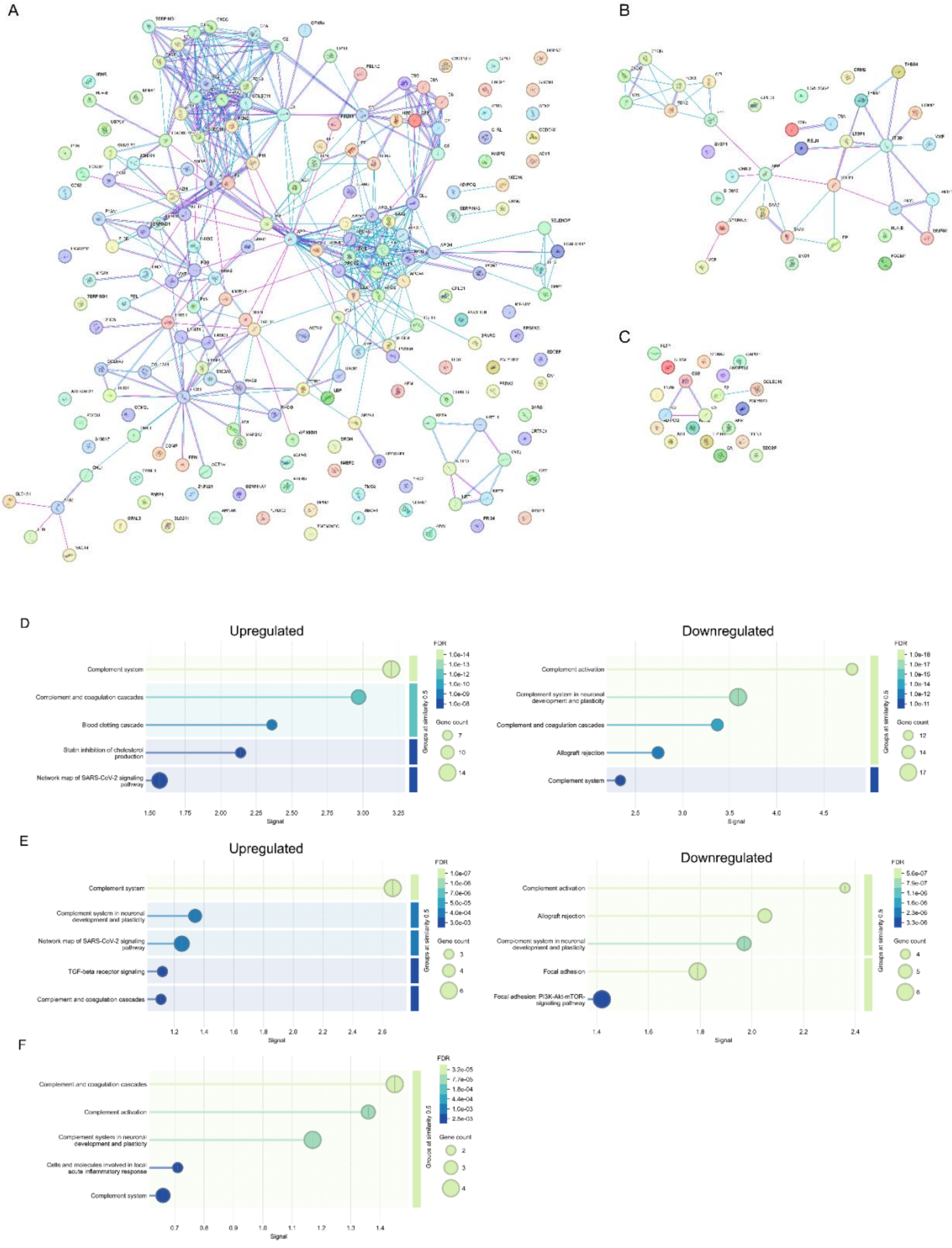
A
B
C
D
Upregulated
Complement system
Complement and coagulation cascades
Blood clotting cascade
Statin inhibition of cholesterol production
Network map of SARS-CoV-2 signaling pathway
Signal
FDR
Gene count
Groups at similarity 0.5
Downregulated
Complement activation
Complement system in neuronal development and plasticity
Complement and coagulation cascades
Allograft rejection
Complement system
E
Upregulated
Complement system
Complement system in neuronal development and plasticity
Network map of SARS-CoV-2 signaling pathway
TGF-beta receptor signaling
Complement and coagulation cascades
Downregulated
Complement activation
Allograft rejection
Complement system in neuronal development and plasticity
Focal adhesion
Focal adhesion: PI3K-Akt-mTOR-signaling pathway
F
Complement and coagulation cascades
Complement activation
Complement system in neuronal development and plasticity
Cells and molecules involved in local acute inflammatory response
Complement system

**Supplementary Figure 2. Functional pathway analysis of differentially abundant sEVs proteins identifies pathways known to be altered in glioma. Related to Figure 4 and Figure 7.**

Protein association network analysis for the **A**) differentially abundant proteins identified between healthy volunteer and glioma patient-derived sEVs in the test cohort (n=305, q<0.05), and for the **B**) concordant (n=35) and **C**) discordant (n=20) proteins (q<0.05 and fold change >±1.5 in the test cohort) between the test and validation cohorts respectively. WikiPathways enrichment analysis of the **D**) differentially abundant proteins identified between healthy volunteer and glioma patient-derived sEVs in the test cohort (n=305, q<0.05) and for the **E**) concordant (n=35) and **F**) discordant (n=20) proteins (q<0.05 and fold change >±1.5 in the test cohort) between the test and validation cohorts respectively, highlighting up to the top 5 upregulated and up to the top 5 downregulated pathways.

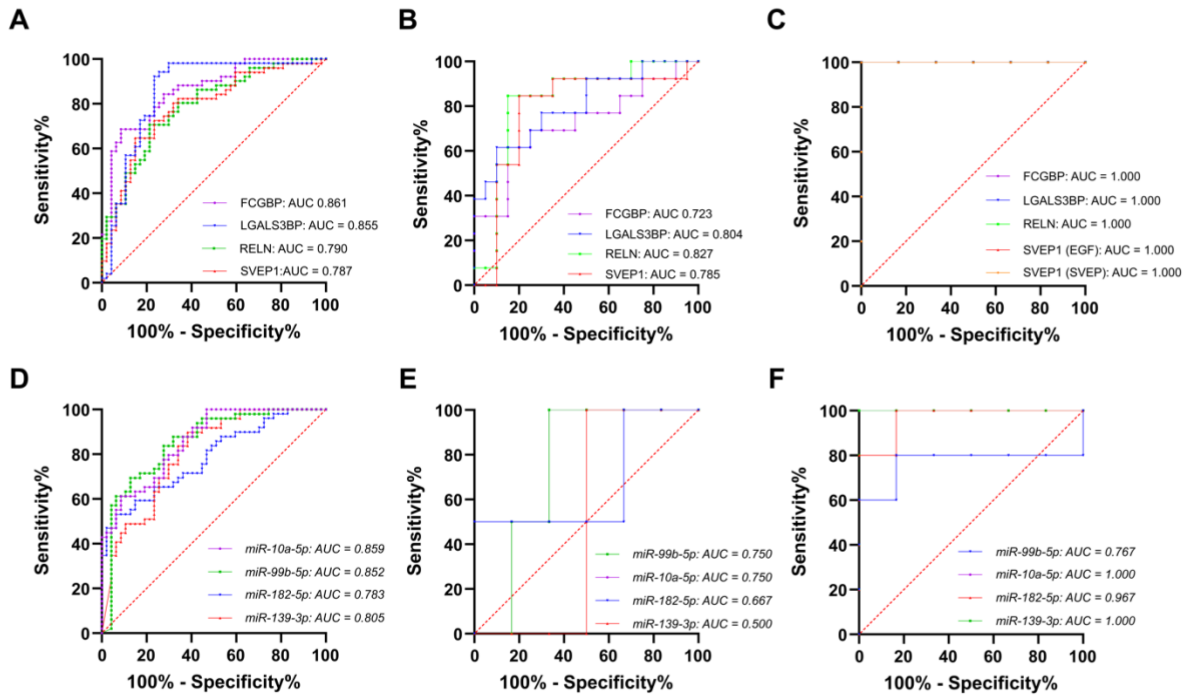


**Supplementary Figure 3. ROC curves for the top performing biomarkers. Related to Figure 4 and Figure 5.**

ROC curves for the top performing protein biomarkers across the: **A**) Test cohort, **B**) Validation cohort, and the **C**) Longitudinal cohort. ROC curves for the top performing microRNA biomarkers across the: **D**) Test cohort, **E**) Validation cohort, and the **F**) Longitudinal cohort.

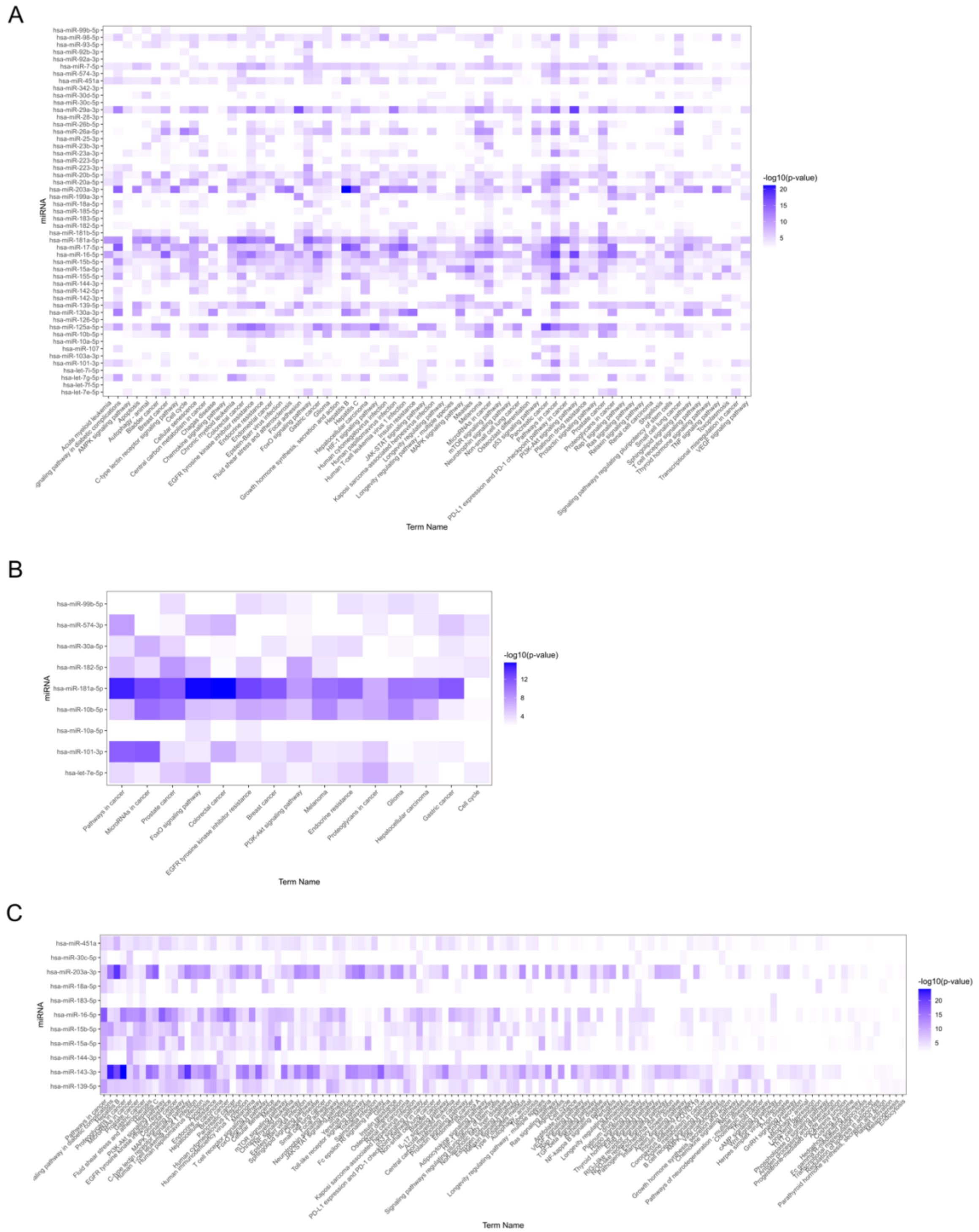
A
miRNA
Term Name
-log10(p-value)
20
15
10
5
hsa-miR-99b-5p
hsa-miR-98-5p
hsa-miR-93-5p
hsa-miR-92b-3p
hsa-miR-92a-3p
hsa-miR-7-5p
hsa-miR-574-3p
hsa-miR-451a
hsa-miR-342-3p
hsa-miR-30d-5p
hsa-miR-30c-5p
hsa-miR-29a-3p
hsa-miR-28-3p
hsa-miR-26b-5p
hsa-miR-26a-5p
hsa-miR-25-3p
hsa-miR-23b-3p
hsa-miR-23a-3p
hsa-miR-223-5p
hsa-miR-223-3p
hsa-miR-20b-5p
hsa-miR-20a-5p
hsa-miR-203a-3p
hsa-miR-199a-3p
hsa-miR-18a-5p
hsa-miR-185-5p
hsa-miR-183-5p
hsa-miR-182-5p
hsa-miR-181b-5p
hsa-miR-181a-5p
hsa-miR-17-5p
hsa-miR-16-5p
hsa-miR-15b-5p
hsa-miR-15a-5p
hsa-miR-155-5p
hsa-miR-144-3p
hsa-miR-142-5p
hsa-miR-142-3p
hsa-miR-139-5p
hsa-miR-130a-3p
hsa-miR-126-5p
hsa-miR-125a-5p
hsa-miR-10b-5p
hsa-miR-10a-5p
hsa-miR-107
hsa-miR-103a-3p
hsa-miR-101-3p
hsa-let-7i-5p
hsa-let-7g-5p
hsa-let-7f-5p
hsa-let-7e-5p
B
miRNA
Term Name
-log10(p-value)
12
8
4
hsa-miR-99b-5p
hsa-miR-574-3p
hsa-miR-30a-5p
hsa-miR-182-5p
hsa-miR-181a-5p
hsa-miR-10b-5p
hsa-miR-10a-5p
hsa-miR-101-3p
hsa-let-7e-5p
Pathways in cancer
MicroRNAs in cancer
Prostate cancer
FoxO signaling pathway
Colorectal cancer
EGFR tyrosine kinase inhibitor resistance
Breast cancer
PI3K-Akt signaling pathway
Melanoma
Endocrine resistance
Proteoglycans in cancer
Glioma
Hepatocellular carcinoma
Gastric cancer
Cell cycle
C
miRNA
Term Name
-log10(p-value)
20
15
10
5
hsa-miR-451a
hsa-miR-30c-5p
hsa-miR-203a-3p
hsa-miR-18a-5p
hsa-miR-183-5p
hsa-miR-16-5p
hsa-miR-15b-5p
hsa-miR-15a-5p
hsa-miR-144-3p
hsa-miR-143-3p
hsa-miR-139-5p

**Supplementary Figure 4. KEGG pathway analysis of differentially expressed sEVs microRNA identifies pathways known to be altered in glioma. Related to Figure 5 and Figure 7.**

**A**) Analysis of the differentially expressed microRNA identified between healthy volunteer and glioma patient-derived sEVs (n=77, adjusted $p<0.05$). Analysis of the **B**) concordant (n=10) and **C**) discordant (n=17) microRNA (adjusted $p<0.05$ and fold change $>\pm1.5$ in the test cohort) between the test and validation cohorts. Heatmaps highlighting pathways common to > 20% identified microRNA and confirmed with strong experimental evidence using DIANA-miRPath v4.0 (miRPathv4)[79].

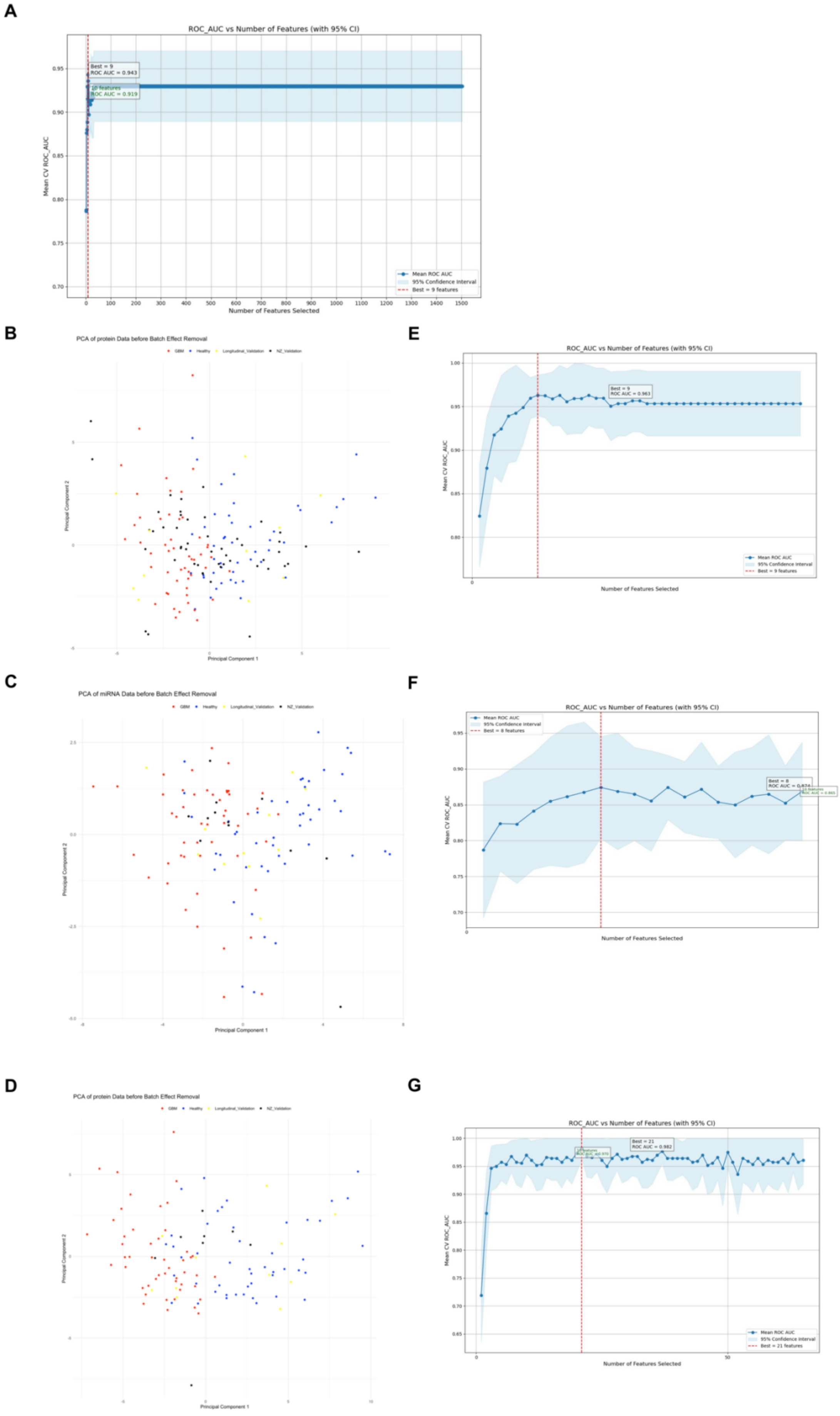

A
ROC_AUC vs Number of Features (with 95% CI)
Best = 9
ROC AUC = 0.943
10 features
ROC AUC = 0.919
Mean CV ROC_AUC
Number of Features Selected
Mean ROC AUC
95% Confidence Interval
Best = 9 features
B
PCA of protein Data before Batch Effect Removal
GBM
Healthy
Longitudinal_Validation
NZ_Validation
Principal Component 1
Principal Component 2
C
PCA of miRNA Data before Batch Effect Removal
GBM
Healthy
Longitudinal_Validation
NZ_Validation
Principal Component 1
Principal Component 2
D
PCA of protein Data before Batch Effect Removal
GBM
Healthy
Longitudinal_Validation
NZ_Validation
Principal Component 1
Principal Component 2
E
ROC_AUC vs Number of Features (with 95% CI)
Best = 9
ROC AUC = 0.963
Mean CV ROC_AUC
Number of Features Selected
Mean ROC AUC
95% Confidence Interval
Best = 9 features
F
ROC_AUC vs Number of Features (with 95% CI)
Mean ROC AUC
95% Confidence Interval
Best = 8 features
Best = 8
Mean CV ROC_AUC
Number of Features Selected
G
ROC_AUC vs Number of Features (with 95% CI)
Best = 21
ROC AUC = 0.982
Mean CV ROC_AUC
Number of Features Selected
Mean ROC AUC
95% Confidence Interval
Best = 21 features

**Supplementary Figure 5. Assessment of the optimal number of features to be included within the developed models alongside principal component analysis demonstrating good overlap of the different cohorts with no clear clustering of samples identified. Related to Figure 6.**

**A**) Analysis of the optimal number of features to be included within the developed model assessed by change in AUC of the ROC analysis for the sEV ATR-FTIR spectral data. Principal component analysis of the z-score values across the three cohorts for the **B**) sEV protein dataset, **C**) sEV microRNA dataset, and **D**) combined sEV protein and microRNA dataset. Analysis of the optimal number of features to be included within the developed model assessed by change in AUC of the ROC analysis for the **E**) sEV protein dataset, **F**) the sEV microRNA dataset, and **G**) the combined dataset.

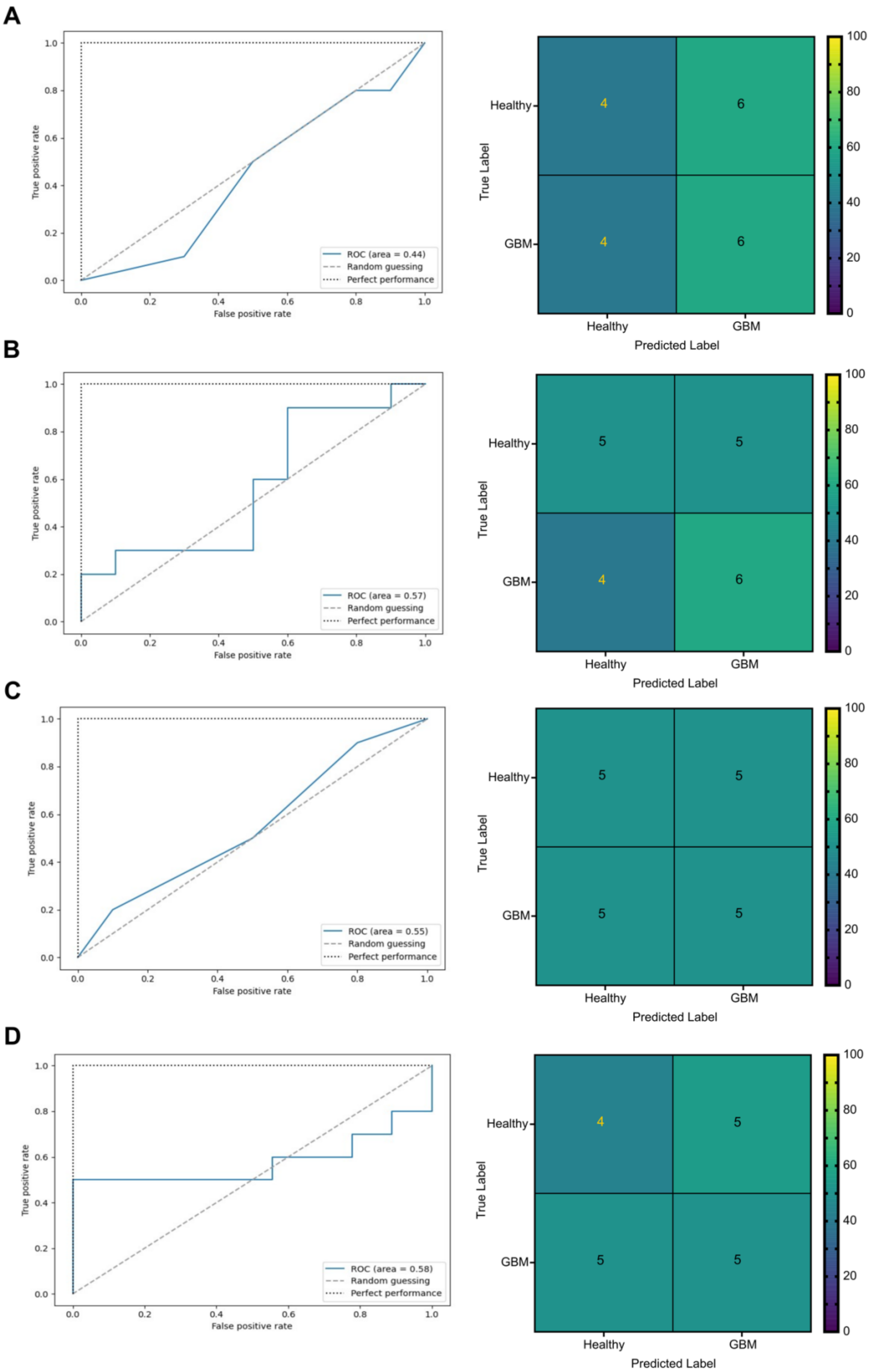

A
ROC (area = 0.44)
Random guessing
Perfect performance
True positive rate
False positive rate
Healthy
GBM
True Label
Predicted Label
4
6
4
6
B
ROC (area = 0.57)
Random guessing
Perfect performance
True positive rate
False positive rate
Healthy
GBM
True Label
Predicted Label
5
5
4
6
C
ROC (area = 0.55)
Random guessing
Perfect performance
True positive rate
False positive rate
Healthy
GBM
True Label
Predicted Label
5
5
5
5
D
ROC (area = 0.58)
Random guessing
Perfect performance
True positive rate
False positive rate
Healthy
GBM
True Label
Predicted Label
4
5
5
5

**Supplementary Figure 6. Class shuffling analysis of the generated machine learning models demonstrated minimal ability to discriminate between glioma patients and healthy volunteers highlighting that the model was not over-fitted and effectively identified glioma patients by leveraging biologically relevant patterns. Related to Figure 6.**

Each panel displays the receiver operating characteristic curve for the performance of the generated model for the class shuffled training sets in terms of the area under the curve and the confusion matrix of the assessment of the respective best model in the test set for the model developed based on the: A) sEV ATR-FTIR spectra (XGBoost, n=10 glioma, n=10 control), B) sEV protein (XGBoost, n=10 glioma, n=10 control), C) sEV microRNA (kNN, n=10 glioma, n=10 control) and D) combined sEV protein and microRNA (RF, n=10 glioma, n=9 control).
